# Chemosensory Profile of South Tyrolean Pinot Blanc Wines: A Multivariate Regression Approach

**DOI:** 10.3390/molecules26206245

**Published:** 2021-10-15

**Authors:** Simone Poggesi, Amanda Dupas de Matos, Edoardo Longo, Danila Chiotti, Ulrich Pedri, Daniela Eisenstecken, Peter Robatscher, Emanuele Boselli

**Affiliations:** 1 Faculty of Science and Technology, Free University of Bozen-Bolzano, Piazza Università 5, 39100 Bolzano, Italy; simone.poggesi@natec.unibz.it (S.P.); emanuele.boselli@unibz.it (E.B.); 2 Oenolab, NOI Techpark, Via Alessandro Volta 13, 39100 Bolzano, Italy; 3 Food Experience and Sensory Testing (Feast) Lab, Massey University, Palmerston North 4410, New Zealand; a.dupasdematos@massey.ac.nz; 4 Riddet Institute, Massey University, Palmerston North 4410, New Zealand; 5 Laimburg Research Centre, Laimburg 6, Pfatten (Vadena), 39040 Auer, Italy; danila.chiotti@laimburg.it (D.C.); ulrich.pedri@laimburg.it (U.P.); daniela.eisenstecken@laimburg.it (D.E.); peter.robatscher@laimburg.it (P.R.)

**Keywords:** pinot blanc, sensory analysis, volatile profile, phenolic profile, multivariate regression, winemaking

## Abstract

A multivariate regression approach based on sensory data and chemical compositions has been applied to study the correlation between the sensory and chemical properties of Pinot Blanc wines from South Tyrol. The sensory properties were identified by descriptive analysis and the chemical profile was obtained by HS-SPME-GC/MS and HPLC. The profiles of the most influencing (positively or negatively) chemical components have been presented for each sensory descriptor. Partial Least Square Regression (PLS) and Principal Component Regression (PCR) models have been tested and applied. Visual (clarity, yellow colour), gustatory (sweetness, sourness, saltiness, bitterness, astringency, and warmness) and olfactory (overall intensity, floral, apple, pear, tropical fruit, dried fruit, fresh vegetative, spicy, cleanness, and off-odours) descriptors have been correlated with the volatile and phenolic profiles, respectively. Each olfactory descriptor was correlated via a PCR model to the volatile compounds, whereas a comprehensive PLS2 regression model was built for the correlation between visual/gustatory descriptors and the phenolic fingerprint. “Apple” was the olfactory descriptor best modelled by PCR, with an adjusted R^2^ of 0.72, with only 20% of the validation samples falling out of the confidence interval (α = 95%). A PLS2 with 6 factors was chosen as the best model for gustatory and visual descriptors related to the phenolic compounds. Finally, the overall quality judgment could be explained by a combination of the calibrated sensory descriptors through a PLS model. This allowed the identification of sensory descriptors such as “olfactory intensity”, “warmness”, “apple”, “saltiness”, “astringency”, “cleanness”, “clarity” and “pear”, which relevantly contributed to the overall quality of Pinot Blanc wines from South Tyrol, obtained with two different winemaking processes and aged in bottle for 18 months.

## 1. Introduction

Pinot Blanc is a versatile, non-aromatic grape variety widely cultivated across the world. Characterized by high titratable acidity, Pinot Blanc can be used for the production of both still and sparkling wines. In Italy, Pinot Blanc is suitably cultivated in areas such as Trentino-South Tyrol, Veneto, Piemonte, Friuli-Venezia Giulia, and Puglia [1], although it is also admitted for DOP and IGP productions throughout the country. In particular, the cultivated area of Pinot Blanc in South Tyrol corresponds to about 600 ha, which represents about 10% of the total viticulture surface area of the province [2].

The aromatic profile of wines can derive from grapes (varietal or primary aromas), production operations such as crushing, pressing, or maceration (secondary aromas), alcoholic and malolactic fermentations (fermentation aromas), and aging processes (maturation aromas) [3,4,5]. In addition to the chemical complexity deriving from the release of the compounds most readily extracted from grapes during crushing, maceration processes can induce the further release of aroma-impacting compounds. The maceration process aims at obtaining the maximum intensity of varietal aroma [6] through the extraction of terpenes, esters, aldehydes and alcohols. Besides, the maceration can increase the extraction of other molecules, such as phenolic compounds [7,8,9,10].

Despite its widespread national and international production, only a few recent studies on the volatile and sensory profiles of Pinot Blanc wines are available in the literature. The aroma of Austrian Pinot Blanc wines was studied to characterize their typical sensory traits [11]. The authors associated the “pear”aroma to the particular typicity of Austrian Pinot Blanc wines; this aroma was associated with short- and long-chain esters, such as ethyl (*E, Z*)-2,4-decadienoate and methyl (*E*)-geranoate [12]. These volatile compounds were mainly associated with notes of apple, banana, apricot, citrus, caramel, and green notes, occasionally with quince and exotic fruits, and in some cases with spicy and nutty notes. When Pinot Blanc wines have matured, they show fresh bread notes, which gain in structure during the aging in-bottle [12]. Recently, Dupas de Matos et al. compared Pinot Blanc wines obtained with an experimental vinification versus a control vinification, in order to highlight the most notable differences in terms of their quality and evolution in-bottle [13]. These authors showed that the Pinot Blanc control wines evolved faster than the experimental wines (differing from the control wines as the experimental winemaking also involved a pre-fermentative cold maceration, addition of pectolytic enzymes, addition of yeast autolysate and a bentonite clarification step) when monitoring the profile of phenolic and volatile compounds as well as the sensory properties.

Many studies have demonstrated the existence of interactions between the evolution of sensory characteristics and the chemical compounds [14]. Explaining the perceptions in terms of cross-modal interactions occurring between aroma and taste is still a challenge [15]. Therefore, to explore these interactions, the best approach would be to move away from a univariate description evaluating the strongest correlations between sensory attributes and chemical compounds taken one-at-a-time, and to study a multivariate approach (where sensory and volatile/non-volatile analyses are combined to calculate models, including interactions), instead. This would result in considering the chemical profile as a whole in its effect on the sensory descriptors.

The present study aims at correlating sensory descriptors and chemical compounds (volatile and phenolic) of two Pinot Blanc wines, by applying two alternative multivariate approaches, namely Partial Least Square Regression (PLS) and Principal Component Regression (PCR). A significant application of multivariate regression, in order to enhance the prediction of the sensory profile of wine based on the chemical fingerprints (not just specific chemicals), was presented to highlight positive or potentially undesired/negative processes occurring during the storage. Precision enology is, in fact, the most innovative tool for the winemaker to reduce losses and enhance the commercial quality of the production. In this sense, positive sensory features could be eventually sought through parallel sensory analysis and instrumental techniques as an analytical support.

## 2. Results

### 2.1. Statistical Analysis Approach

For the statistical analysis, multivariate regression approaches were optimized and applied on three different datasets: (i) overall quality judgment and sensory descriptors, (ii) GC/MS data and olfactory descriptors, and (iii) HPLC data and visual and gustatory descriptors. This arrangement of the study was imposed after considering the parameters evaluated by the trained panel (visual, olfactory, and gustatory aspects, respectively).

In the sensory dataset (*i*), a PLS1 model was built considering the overall quality judgment (objective assessment) as y-vector and the sensory descriptors as the X-matrix. In the GC/MS dataset (*ii*), repeated PLS1 were performed on each sensory descriptor as the y-dependent variable and the aroma compounds as the X-matrix. Alternatively, a unique PLS2 model was computed on all sensory descriptors as the Y-matrix and the aromas compounds as the X-matrix, to study the correlations between volatile compounds and sensory variables and their interaction.

In the HPLC dataset (*iii*), a unique PLS2 model was applied to the sensory (gustatory and visual) descriptors as the Y-matrix and non-volatile compounds as X-matrix. For the GC-MS and HPLC data, PCR was computed with sensory data as Y-matrix and volatile compounds as X-matrix. The HPLC and GC matrices were reconstructed with a Nonlinear Iterative Partial Least Square (NIPALS) algorithm for predicting missing value in a PCA model [16].

**Figure 1:**
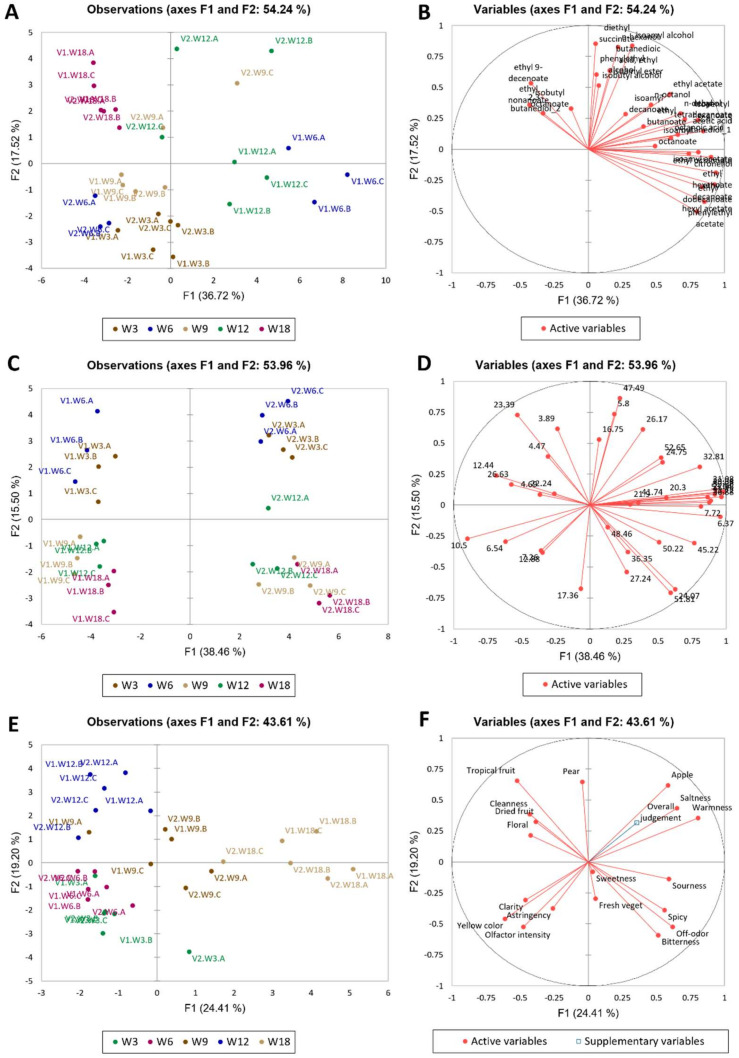
Principal component analysis for the GC-MS data ((**A**) observation plot and (**B**) variables plot), HPLC data ((**C**) observation plot and (**D**) variables plot), and sensory data € observation plot and (**F**) variables plot). W: wine; 3, 6, 9, 12,18: months of storage in bottle; V1: control vinification, V2: experimental vinification. Overall quality judgment was used as a supplementary (non-active) variable in the analysis.

In order to qualitatively explore the main variability sources of the model, the data have been initially studied using Principal Component Analysis (PCA) (Figure 1). Notably, the results from 3 to 9 months of storage in bottles were discussed in a previous paper [13] and have now been integrated up to 18 months of storage. Samples after 12 and 18 months of storage were interesting, as certain relevant parameters such as the measured overall quality judgment (supplementary variable projected onto the PC1 vs. PC2 loadings plot, Figure 1E,F) resulted higher at month 18 (W18 samples), although not significantly, and in particular for V1 (control vinification).

Considering the volatile compounds (Figure 1A,B), the PCA models showed clusters by month and wine for most triplicates, although a particular trend over time or vinification could not be clearly observed for all triplets along any particular direction in the PC1 vs. PC2 Score plot. However, W18 samples were completely clustered at low PC1 and high PC2 values, indicating also that some “re-convergence” of V1 and V2 volatile profiles took place after the biggest spreading at month 6 (W6) and 12 (W12). To simplify the interpretation, considering particularly the direction separating W3 from W18, only some of the compounds of the volatile profile characterized the temporal evolution, e.g., ethyl 9-decenoate, 2,3-butanediol, isobutyl octanoate, ethyl nonanoate, diethyl succinate, isoamyl alcohol, phenylethyl alcohol and n-hexanol.

Regarding the phenolic profile (Figure 1C,D), here the trends were clearly observable. PC1 separated neatly the two vinifications, whereas PC2 mostly represented temporal evolutions for both wines, as W3-6 were separated in PC2 from W9-18 for both wines along PC2 (the evolution proceeded from high to low score values). The variables in the loadings plot are reported as retention times, as many compounds could not be definitively and unambiguously identified by LC-MS analysis.

A consistent temporal trend was not observed for either of the sensory profiles (Figure 1E,F), when considering the entire time range. To simplify the interpretation, when considering the differences between W3 and W18, the overall evolution mainly involved the “apple”, “saltiness”, “warmness”, which increased overall and “yellow colour”, “olfactory intensity”, “astringency” and “clarity”, which decreased from W3 to W18 only. Interestingly, the overall quality judgement (applied as supporting variable, projected onto the PC1 vs. PC2 subspace) was higher at W18 than W3 and, and, at W18, it was higher in V1 than V2.

Successively, one-way or two-way ANOVA (considering interactions) has been applied to study the non-volatile phenols, the volatile compounds and the sensory profiles, to provide a more in-depth explanation of the PCA. To simplify, only the variables significant at α = 0.05 are shown. Tables S1 and S2 (supplementary materials) show the one-way ANOVA for the phenols on Time and Wine, respectively. The most significant variables (<0.0001) for the ANOVA on Time were 3.89, 4.47, 4.69, 5.80, 21.9, 23.39, 36.35, 47.49, 48.46 and for the ANOVA on Wine were 6.37, 7.72, 10.5, 12.44, 27.95, 29.34, 31.08, 32.81, 33.72, 36.88, 39.95, 45.22, 49.63, 53.04. Instead, Table S3 shows the two-way ANOVA with interaction Time*Wine; this model shows that almost all the variables were significant; only 17.36, 20.3, and 52.65 were significant.

Table S4 shows the one-way ANOVA for the volatile compounds on the Time. The most significant variables (<0.0001) were diethyl succinate and phenylethyl acetate. Table S5 shows the one-way ANOVA for the volatile compounds in the wine; the variables that show significant differences (<0.05) were isobutyl octanoate, 2,3-butanediol (isomer 2), and octanoic acid. The Table S6 shows the two-way ANOVA with interaction Time*Wine on volatile compounds. In this case, many different variables showed significant differences (<0.0001) (ethyl acetate, isoamyl acetate, isoamyl alcohol, ethyl hexanoate, hexyl acetate, n-hexanol, ethyl octanoate, acetic acid, isopentyl hexanoate, ethyl nonanoate, 2,3 butanediol (isomer 2), ethyl decanoate, diethyl succinate, phenyl ethyl acetate, ethyl dodecanoate, and octanoic acid). One-way and two-way ANOVA was also computed for the sensory descriptors. Table S7 shows the one-way ANOVA for the sensory descriptors on the Time. The most significant variables (<0.0001) are yellow colour, olfactory intensity, apple, tropical fruit, warmness, sweetness, sourness, saltiness, bitterness. The one-way ANOVA on Wine did not show any significant variable (data not shown). Finally, a two-way ANOVA reporting the interactions between Time*Wine showed that the most significant variables were yellow colour, olfactory intensity, tropical fruit, warmness, saltiness, and bitterness.

### 2.2. Volatile Compounds

The volatile compounds (tentative identification), the respective Retention Time (RT, min), and the calculated Retention Index (RI) were reported in Table S9.

**Figure 2:**
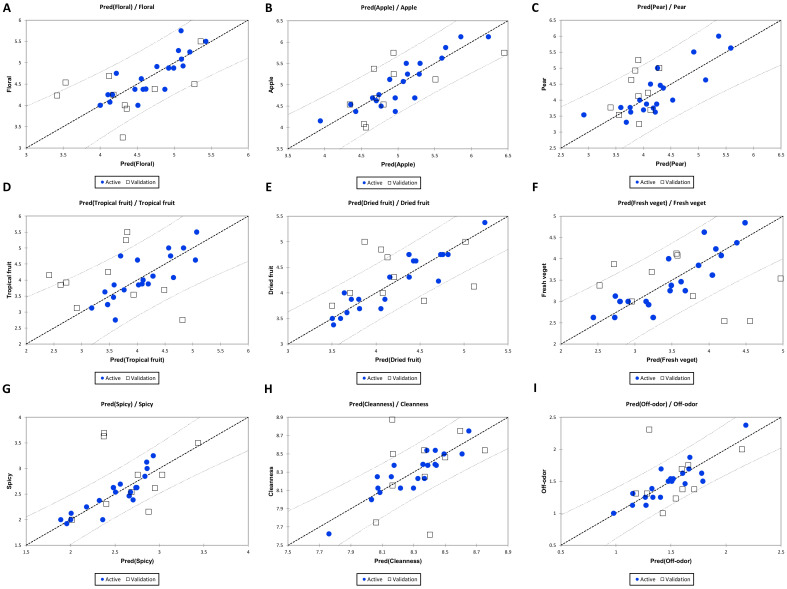
Predicted/observed regression plots calculated on the PCR on volatile compounds (the confidence interval is set at 95%) for olfactory descriptors: (**A**) Floral; (**B**) Apple; (**C**) Pear; (**D**) Tropical fruit; (**E**) Dried fruit; (**F**) Fresh veget.; (**G**) Spicy; (**H**) Cleanness; (**I**) Off-odor.

Figure 2 shows the predicted/observed regression graphs obtained by PCR. Specifically, ŷ vs. y plots show that at most 20% of samples of the validation sets (taken after observations randomization) for floral (A), apple (B), and off-odour (I) descriptors are outside the computed confidence intervals (α = 95%). For tropical fruit (D), dried fruit (E), fresh vegetative (F), and cleanness (F), 40% of the validation set is outside the confidence bands. For spicy (G), 30% of the validation set is outside the 95% confidence bands. Pear descriptor (C) showed the best result in validation, as only 10% of the validation set is outside of the confidence interval. The same descriptors were also evaluated on the aroma dataset with individual PLS1 models, which showed worse results in prediction than in PCR (data not shown). To evaluate globally the performance in calibration, adjusted $\hat{R}$^2^ and RMSE for the different aroma sensory descriptors’ models obtained by PCR are reported in Table 1.

As shown in Table 1, the adjusted R^2^ for each descriptor is higher than 0.5, except for tropical fruit, while RMSE, which represents the residuals or prediction error in the model, is always below 0.5 except for pear and tropical fruit aromas.

**Figure 3:**
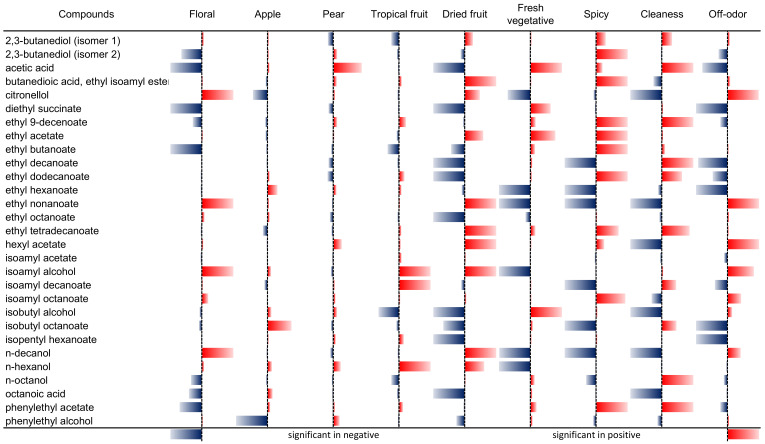
Summary of the calculated *p*-values and standardized coefficients of the volatile compounds in the regression models. The absolute length of the bars represents the significance expressed as “1/*p*-value”; consequently, the longer the bar is, the more significant is the effect of that compound. The colour and the direction of the bar indicate instead the sign of the standardized coefficient in the respective regression equation: Red = positive sign/correlation, Blue = negative sign/correlation.

Figure 3 summarizes the overall effect of each aroma compound to each sensory descriptor, included as the impact of each term/compound on the related regression equation. The colour and sign of the terms reflect the correlation of the related coefficient (red for positive correlations, blue for negative correlations), whereas the bar absolute length is proportional to the inverse of the associated calculated *p*-value (1/*p*-val.). Consequently, the smaller the *p*-value is, the bigger is the related bar.

The standardized coefficients and standardized residuals are shown in Figure S2. The standardized coefficients are presented in statistical significance order, with the different zone divided by a dotted line indicating a different level of significance. The first indicates a significance critical value (crit. *p*-value) < 0.001, the second (where present) indicates a *p*-value < 0.01 and the third (if is present) < 0.05, respectively. These coefficients represent the calculated regression model coefficients, with the associated confidence intervals. Each sensory descriptor could be therefore associated with a subset of aroma compounds showing a significant or non-significant effect, depending on the associated *p*-value. No interaction nor non-linear terms (among volatile compounds) improved the quality of the calibration model (data not shown). However, it is clear that no unique dependence of a descriptor on just one volatile compound was observed. Instead, a linear combination of several compounds with significant impact was found. All sensory descriptors were therefore expressed as linear combinations (although no effects from interacting or quadratic terms were significant) of subgroups for significant and non-significant aroma compounds.

Interestingly, as shown in Figure 3, four volatile compounds were statistically significant with positive effects for the floral descriptor (citronellol, ethyl nonanoate, isoamyl alcohol, and n-decanol), whereas acetic acid, diethyl succinate, and ethyl butanoate had a negative effect. Only a few volatile compounds were significant for the apple descriptor (negative effect: phenylethyl alcohol, citronellol; positive effect: isobutyl octanoate). The only compound that had a significant effect for the pear descriptor was acetic acid, with a positive effect on the model. Even though the volatile compounds showed lower significance values, they still contributed with a different effect on the pear descriptor. For the tropical fruit, three different volatile compounds showed significant positive effects (*n*-hexanol, isoamyl decanoate, and isoamyl alcohol), whereas isobutyl alcohol showed a significant negative effect on the model.

Interestingly, specific compounds showed a positive effect on cleanness and a negative effect on off-odour, highlighting the opposition between these two descriptors: acetic acid, citronellol, ethyl decanoate, ethyl dodecanoate, isoamyl decanoate, and isobutyl octanoate.

### 2.3. Visual and Gustatory Descriptors

The identification of several compounds had already been presented along with their fragmentation spectra in a previous report on the same Pinot Blanc wines [13]. This tentative identification, reported in Table S10, has been extended and obtained by off-line LC-MS analysis on a set of representative samples across the entire storage time. Peaks were expressed by their retention times, their MS^1^ *m/z* values with an indication of the ionization mode (negative or positive) and the measured spectrum λ_MAX_ (nm) of the peak and the peak area (as Abs/mAU at 280 nm per retention time). The peak table has been aligned manually ensuring that each peak showed the same UV-Vis spectral features. Compounds with single λ_MAX_ at 285–275 nm could be associated tentatively to flavan-3-ols and their oligomers (proanthocyanidins); the spectral profile of dihydroflavonols (flavanonol) with a higher maximun around 290–285 nm and a broad band of lower intensity located around 330 nm was applied to identify taxifolin and astilbin (taxifolin-3-*O*-rhamnetin); simple and derivated cinnamic acids presented clear features as a double maximum of relatively similar intensities between 280 nm and 315 nm (simple cinnamic acids) or around 290 nm and 325 nm (derivatives such as cinnamoyl tartrates e.g., caftaric acid); benzoic acids and their derivatives (e.g., esters) are characterized by λ_MAX_ at 270–260 nm. This classification allowed to suitably restrict the possible assignment of the observed peaks to a specific class.

Using the complete peak list, multivariate regression models were built to explain visual and gustatory sensory descriptors.

**Figure 4:**
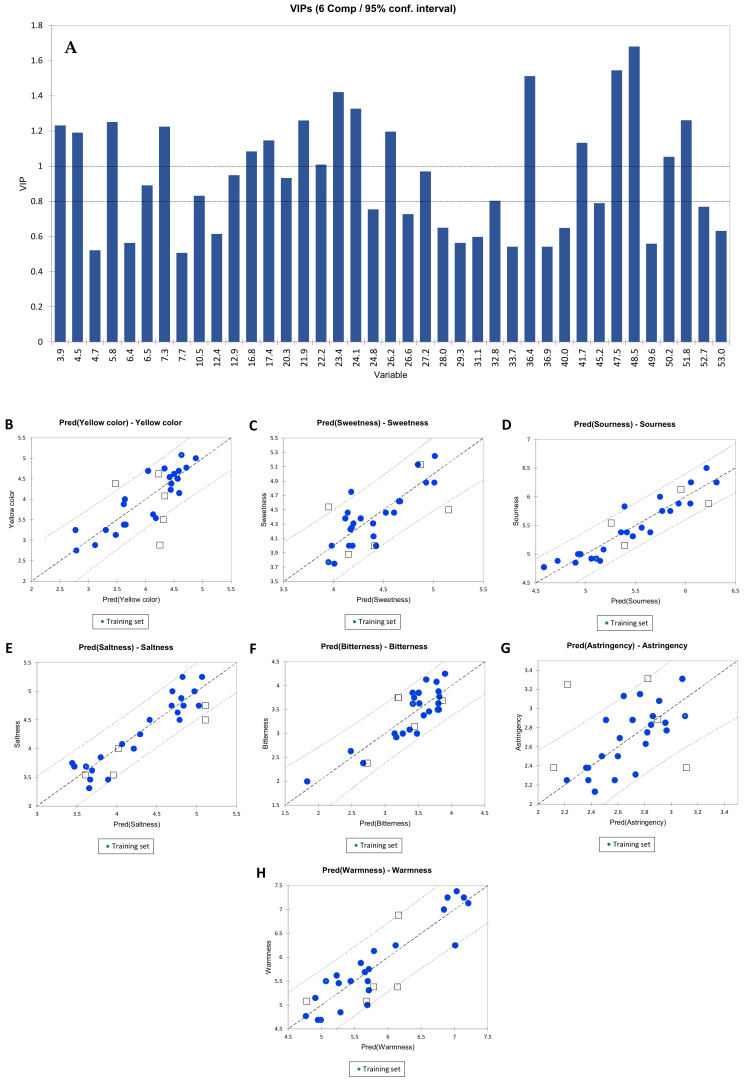
Partial Least Square Regression with multiple dependent variables (PLS2) for gustatory and visual descriptors: (**A**) VIP (Variables Importance in Projection) at the component used for calculating the model; (**B** to **H**) Predicted/observed regression graph for visual and gustatory sensory descriptors (the confidence interval is set at 95%). Numbers of the X-axis are HPLC retention times of phenolics.

PLS2 was chosen as the best approach for gustatory and visual descriptors. The same data were also processed by PCR, which showed a worse prediction than PLS2. In Figure 4, the PLS2 regression for visual and gustatory descriptors is reported (yellow colour, sweetness, sourness, saltiness, bitterness, astringency, and warmness). The best PLS2 regression model was calculated at 6 components and the qualitative index Q^2^(cum), R^2^X (cum), and R^2^Ycum up to 6 components are reported in Table 2. In Figure 4A the VIP. The VIP describes the weight of the variables (HPLC peaks with assigned retention time) in the model; VIP were divided into two categories: the first included all the variables with a score higher than 1.2 (the most significant variables) (48.4, 47.4, 36.3, 23.3, 24, 51.8, 5.8, 21.9, 3.89 and 7.26), and the second category includes all the variables that are between 1.2 and 0.8 (26.1, 4.47, 17.3, 41.7, 16.7, 50.2, 22.2, 27.2, 12.8, 20.3, 6.5, 10.5 and 31.8). The most important phenolic compound was 48.5 (λ_MAX_ = 290 nm, 317 nm), the second most important was 47.5 (λ_MAX_ = 290 nm) and the third one was 36.4 (fertaric acid).

Table 2 reports the quality index Q^2^, R^2^Y, and R^2^X for each PLS2 model calculated at 1 to 6 components. The best PLS model was the one at 6 components, in fact increasing the number of components increased the cumulative Q2, R2X and R2Y indices but worsened the prediction quality. Table 3 shows the R^2^ index, standard deviation, and RMSE with 6 components for the different sensory descriptors. These indices, in combination with the indices reported in Table 2, were used to select the best model.

In Figure S3, the PLS2 standardized coefficients plots for the visual and gustatory descriptors are reported. Figure 4B–G shows the regression graph for the different visual and gustatory sensory descriptors. As described in the Methods section, seven randomly extracted samples were chosen as the validation set in the PLS2 model. In order to have a predictive model, it is relevant that the validation sets are within the confidence interval (95%). Specifically, Figure 4B shows the regression for the yellow colour descriptor, which shows that 20% of the validation set outside of the confidence interval fix it at 95%. Also the warmness is shown in Figure 4H with just 10% of validation set outside of the confidence interval. It is interesting to notice that the model for bitterness (Figure 4F) does not have outliers in validation. Instead, Figure 4C shows the regression for sweetness, which had 20% of the validation set outside of the confidence intervals. Figure 4G shows the regression for astringency that has 20% of the validation set outside of the confidence interval.

**Figure 5:**
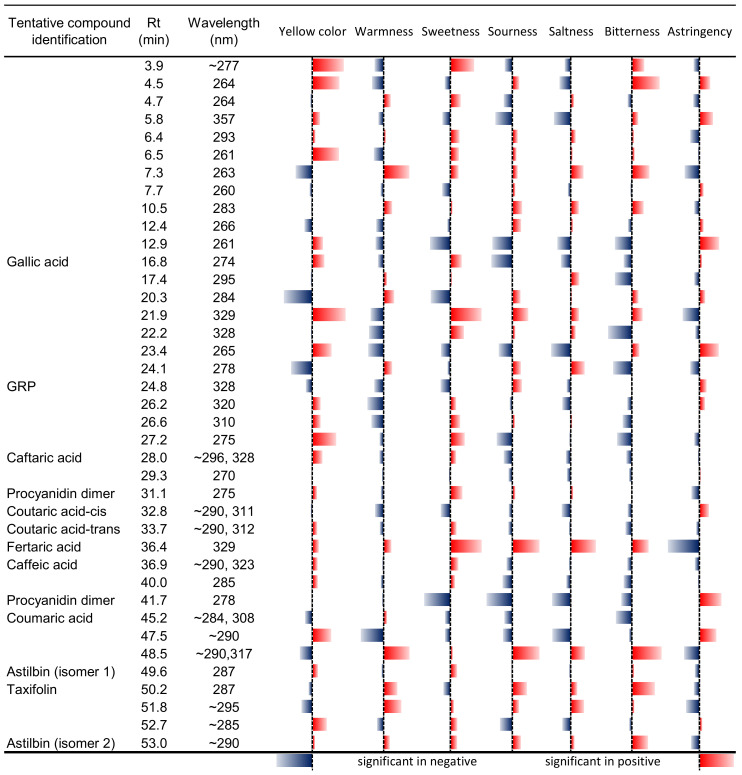
Summary of the calculated *p*-values and standardized coefficients of the phenolic compounds in the regression models. The absolute length of the bars represents the significance expressed as “1/*p*-value”; consequently, the longer the bar is, the more significant is the effect of that compound. The colour and the direction of the bar indicate instead the sign of the standardized coefficient in the respective regression equation: Red = positive sign/correlation, blue = negative sign/correlation.

Figure 5 shows the summary for calculated *p*-value and standardized coefficients of the volatile compound in the regression models, as shown in Figure 3. The compounds 12.9 (λ_MAX_ = 261 nm) and 16.8 (gallic acid) showed a similarity for yellow colour intensity, warmness, sourness, saltiness, bitterness, and astringency, except for sweetness. The compound 36.4 (fertaric acid) showed a positive effect for yellow colour intensity, warmness, sweetness, sourness, and bitterness except for astringency. As expected, compound 41.7 (a procyanidin dimer, λ_MAX_ = 278 nm) showed a significant negative effect except for astringency. This contrary effect was also shown by the 47.5 (λ_MAX_ = 290 nm) and 48.5 (λ_MAX_ = 290 nm and 317 nm) compounds. The 47.5 (λ_MAX_ = 290 nm) compound showed a positive effect in yellow colour intensity and astringency, and a negative effect on the other descriptors. Instead, the 48.5 (λ_MAX_ = 290 nm and 317 nm) compound showed a negative effect for yellow colour and astringency but a positive effect for warmness, sweetness, sourness, saltiness, and bitterness.

### 2.4. Overall Quality Judgment

**Figure 6:**
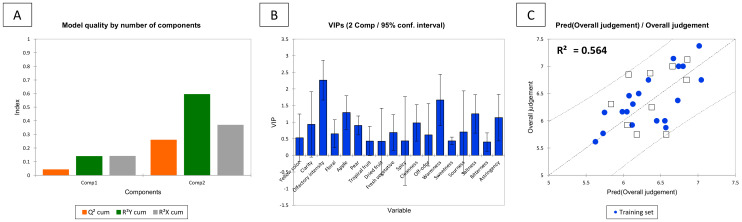
Summary of the partial least square regression for the overall quality judgment over the sensory descriptors. (**A**) Histogram of model quality by the number of components, Q2, R^2^Y, and R^2^X indexes; (**B**) VIPs calculated at two components for all the sensory variables; (**C**) observed values vs. predicted values of overall quality judgment.

The ‘overall quality judgment’ is a variable dependent on the sensory descriptors defined by the panel. The resulting chosen PLS model (2 factors) showed a complex and multidimensional variability. The model with two factors had a Q^2^(cum) of 0.26 considering all the variables, and R^2^X (cum) of 0.37, and R^2^Y (cum) of 0.59 (Figure 6A). The VIPs graph (Figure 6B) allowed to outline which variables had more influence on the model of the overall quality (arbitrarily with a VIP > 0.8). The most important variable was the olfactory intensity (2.26), followed by warmness (1.66), apple (1.28), saltiness (1.24), astringency (1.13), cleanness (0.97), clarity (0.93) and pear (0.90). On the other hand, the model showed that the most important variables were the ones strictly correlated with the aroma profile, as the highest VIPs are olfactory intensity and cleanness. It is interesting to note also that the specific aroma descriptors “apple” and “pear”, as observed also by Philipp et al. (2018), have been found to be relevant as variables (VIP) for the overall quality of these Pinot Blanc wines.

## 3. Discussion

The traditional definition of quality is the identification of defects and deviations from an ideal product. The principal challenge in expressing quality is to provide an overall score, despite all the multidimensional differences featured by the product [17]. It is commonly established that quality is a complex and multidimensional concept, containing both subjective and objective components, which are situation-specific, fluid and dynamic across time [18,19]. The accurate assessment of the sensory quality is a prerequisite for identifying deviations from sensory requirements/specifications and for the application of the appropriate corrective actions [20]. This is particularly important in winemaking practices, where the decision-making process is often left to the emotional and personal choices of the winemaker, which are based on subjective wine-tasting protocols. Many traditional quality evaluation methods for food products are instead defect-oriented and based on the use of expert assessors [21,22]. In this study, the overall quality judgment has been described in terms of sensory descriptors previously defined by a trained sensory panel. This showed the advantage of correlating an overall quality parameter upon sensory descriptors and their interactions.

In turn, each sensory descriptor was correlated and explained as a combination of chemical parameters. Our assumption, based on the known physiological mechanisms of sensory perceptions [17], was that the sensory descriptors evaluated orthonasally could be associated with the volatile compounds. Similarly, gustatory and visual descriptors should be mostly affected by the non-volatile compounds.

Accordingly, multivariate regression models were built and, as a result, each sensory descriptor was defined as a linear combination of several significant chemical variables. The overall quality judgment was described as an objective assessment of all sensory features. In this sense, this parameter could support the sensory quality evaluation in terms of chemical composition, although this definition might be limited in its application by (1) the boundaries defined by the sample representativity, including geographical origin and oenological variables, (2) the diversity within the panel, e.g., the individual differences in sensitivity to certain memory of sensations, subjective variability about attributes due to different backgrounds, such as regionality.

Concerning the PCR model, for olfactory descriptors versus volatile compounds, the descriptor that gave the best results in terms of quality was the apple aroma, which showed an adjusted R^2^ of 0.55 and 20% of the validation set outside the confidence bands. Regarding the volatile compounds, the standardized coefficients showed a negative effect on the model equation for phenylethyl alcohol and citronellol and a positive effect for isobutyl octanoate. Philipp et al. [12] showed that the sensory attribute apple in Austrian Pinot Blanc should always be associated with pear aroma, but the PCR model applied to this Pinot Blanc from South Tyrol did not show optimal quality for pear attribute. This might be explained by the fact that the pear and apple aromas have many related compounds in common, i.e., citronellol, isobutyl octanoate and phenylethyl alcohol (acetic acid seemed also to contribute to the range found in this wines), which have a significant opposite effect on these two descriptors. Moreover, these two descriptors were difficult to be distinguished by the panellists and required more training compared to the other sensory descriptors. Therefore, according to the PLS2 model on the visual and gustatory descriptors versus non-volatile compounds, the most important correlations caused synergic or opposite effects for certain compounds on the sensory variables.

In conclusion, this work attempted at creating models of calibrated sensory descriptors on the basis of measured chemical variables and considered these descriptors as the effect of complex chemical profiles. It is important to note that even though the number of testers was relatively low, the models proved to be robust for our set of samples and the chemical variables and sensory descriptors on which they were built were well in agreement with the known quality markers of this wine. In fact, the model built on our datasets did show notable good correlations for specific descriptors of interest. Overall, this study allowed, on one side, to correlate the sensory descriptors with analytical parameters, whose measurement was performed applying standard analytical methodologies, and on the other, to discuss the overall quality score given by the trained panel as an expression of the sensory descriptors themselves.

## 4. Materials and Methods

### 4.1. Sampling and Winemaking

The winemaking procedures to obtain the samples studied in this report have been described previously [13]. Briefly, the Pinot Blanc grapes were harvested in 2018 at Dorf Tirol (South Tyrol, Italy), in a vineyard located at 550 m (a.s.l.) altitude. The winemaking process was performed as described by Dupas de Matos et al. [13]. Shortly, the grapes were mechanically destemmed and the whole mass was divided into three replicates for each of two winemaking procedures (herein called V1 and V2). The grapes for V1 (control wine) were pressed in two steps. Potassium metabisulfite (0.06 g·L^−1^) was added to the must after transferring it to the tank and cold sedimentation was performed to eliminate the deposited residues. The day after, a Zymaflore yeast (Laffort Italia S.r.L., Tortona, Italy) inoculum was prepared. When the alcoholic fermentation was over for V1, the wines were racked and cooled down to 4 °C for tartaric stabilization for 12 d.

For the V2 (experimental wine) the grapes underwent a pre-fermentative cold maceration in a stainless-steel tank with 0.6 g·L^−1^ of pectolytic enzyme (Trenolin frio, Erbslöh, Geisenheim, Germany). All other operations carried out on V1 were also performed on V2, with the exception of the addition of 0.2 ·^−1^ of a yeast extract (B-energia, HTS Enologia, Marsala, Italy) in V2, which was performed six days after the start of alcoholic fermentation.

At the end of alcoholic fermentation, tartaric stabilization was performed after racking (4 °C for 12 days). After two months of storage in stainless steel tank, V2 wines were added with 0.7 g·L^−1^ of bentonite (Nacalit, Poretec, Erbslöh, Geisenheim, Germany). The bottling was performed according to the following procedure: pre-filtration and sterile microfiltration, then the wines were poured into a steel tank and a N_2_ pressure was applied to accelerate the flow of the wines through the filters. Finally, the wines were bottled in 500 mL green glass bottles and closed with a screw cap. Following this procedure, a total of 30 bottles per wine was obtained and were stored at a constant temperature of 16 °C until the opening for the analyses, as shown in Figure S1 (Supplementary materials).

### 4.2. Volatile Profile by HS-SPME-GC/MS

The SPME methods were adapted from the literature [23]. First, 8 mL of wine sample was added into a 20 mL-glass vial with 1 mL of saturated NaCl. Then 50 µL of 2-methyl-3-pentanol (corresponding to 103 mg·L^−1^ final concentration) was added as internal standard (I.S.) solution and closed with a perforable screw-cap. The vial was left for 5 min at 40 °C in a heating bath with continuous stirring at 300 rpm. Afterward, the stirring was stopped and an activated SPME fibre (DVB/CAR/PRDMS, 50/30 µm, 1 cm) was exposed to the headspace of the vial for 30 min under continuous heating (40 °C).

The GC/MS analysis was performed with manual injection on an Agilent 7890A gas chromatograph coupled to an Agilent 5975 quadrupole mass detector. The thermal desorption took place at 240 °C for 6 min. A MEGA-WAX Spirit column (0.30 µm/0.18 mm/40 m; MEGA S.r.l., Legnano, Italy) was used to separate the volatile compounds in split mode (1:10) and a carrier gas (helium) flow rate at 0.7 mg·L^−1^ (constant flow). The oven temperature ramp was 40 °C for 0.2 min, 40–180 °C at 3 °C·min^−1^, then 180–230 °C with a 10 °C·min^−1^ rate, and finally kept for 3 min at 230 °C. The mass spectrometer operated in EI mode at 70 eV. The mass range was 34–360 *m/z* at 1 spectrum·s^−1^; the temperature of the ion source and quadrupole were 230 °C and 150 °C, respectively.

The samples were analysed in a randomized mode whenever possible. GC/MS data integration was performed automatically using the provided software (Chemstation, Agilent, Santa Clara, CA, USA). Total Ion Current (TIC) peaks were expressed as areas vs. retention time and were aligned manually. The compounds were assigned to chemical species by coupling two different approaches: comparison of acquired spectra with reference mass spectra (NIST 2011 database, ref. [24]) and calculating the linear retention indexes (LRI) on the base of the elution series of linear alkanes standards (analytical standards C_7_–C_40_ in dichloromethane, Sigma-Aldrich *plus* the additional injection of pure hexane and pentane). The non-isothermal Linear Retention Indexes (LRI) were calculated to identify the compounds from the reference alkane standards retention times, according to [25].

### 4.3. HPLC-DAD and HPLC-MS Analysis of Non-Volatile Compounds

The non-volatile compounds were analysed by HPLC-DAD as described by [13]. A Eurosphere II C18 (250 × 4.6 mm × 5 µm, Knauer, Lab Service Analytica, Bologna, Italy) stationary phase was used, mounted in a Nexera X2 UPLC system (Shimadzu, Milano, Italy) equipped with a UV-Vis diode array (DAD) and a fluorescence detector (in series). The column was kept at 30 °C. The HPLC mobile phase was solvent A (0.1% formic acid in MilliQ water) and solvent B (0.1% formic acid in acetonitrile). The gradient program was as follows: 1% B for 0–2.5 min, then increased to 25% B until 50 min, then from 25% B to 99% B in 1 min, then at 99% B for 4 min, after that from 99%B back to 1% B in 3 min. The separation was carried at 0.7 mL·min^−1^. The peak integration was calculated automatically by the Labsolution (ver. 5.92) software (Shimadzu, Kyoto, Japan) and the peak alignments were done manually.

HPLC-MS analysis was performed offline on a Ultimate 3000 UHPLC coupled to a TSQ Quantiva QqQ (Thermo Fisher Scientific, Rodano, Milano, Italy) for identification. The method used the same column and the same gradient as described for the HPLC-DAD analysis [13].

### 4.4. Descriptive Sensory Analysis

A panel of eleven subjects (7 males and 5 females, aged 24 ± 5 years old), participated in a 16 hrs sensory training over eight weeks at the Free University of Bozen-Bolzano (Italy). Participants were selected on the basis of their sensory sensitivities and ability to discriminate differences in sensory properties among samples. Informed consent was signed by who agreed to participate in the subsequent sensory analysis sessions, without any monetary reward. The panel received specific training using intensity scales (ISO 8586:2012), which is fully reported in [13]. Before starting each sensory session, panellists received from the panel leader detailed instructions on the definition of the descriptors and how to conduct the sensory evaluation (e.g., order of sampling from the left to the right direction). Panellists were informed that the descriptors would be related to visual evaluation (clarity and yellow colour intensity), olfactory evaluation (olfactory intensity, floral, apple, pear, tropical fruit, dried fruit, spicy, fresh vegetative, cleanness, and off-odour), gustatory evaluation (sweetness, sourness, saltiness, bitterness, astringency, and warmness). In addition, the panel was asked to give an overall quality judgment considering the sensory descriptors themselves. The complete list of descriptors and respective definitions are reported in Table 4. The wine samples were evaluated according to Quantitative Descriptive Analysis (QDA^®^, Tragon Corporation, Arlington, TX, USA) under the conditions described in the UNI 10957:2003 procedure. The wine bottles were opened just before the evaluation and 30 mL of wine per glass at around 16 °C were offered randomly (in triplicates) to the panellists in ISO wine glasses codified with random 3-digit numbers. Panellists were provided with plain crackers without added salt and water to cleanse the palate between samples. The presentation order of the wine samples was randomized between and within participants to avoid bias (complete and balanced design).

### 4.5. Data Analysis

The statistical elaboration was performed with XLSTAT software (Addinsoft, New York, NY, USA). To approach the complexity and aim at obtaining simpler and more accurate regression models, the visual (clarity and yellow colour) and gustatory descriptors (sweetness, sourness, saltiness, bitterness, astringency, and warmness) were elaborated separately from the olfactory descriptors (overall intensity, floral, apple, pear, tropical fruit, dried fruit, fresh vegetative, spicy, cleanness, and off-odours), on the basis that the olfactory descriptors would be affected by volatile compounds only, whereas the visual and gustatory descriptors by non-volatile compounds.

#### 4.5.1. Principal Component Analysis and ANOVA

Principal component analysis (PCA) was used to study the correlation between variables and factors and reduce the dimensionality given by many variables in the dataset. The PCA was computed in three separate datasets (volatile compounds, non-volatile phenols, and sensory descriptors). PCA for volatile compounds and non-volatile phenols were treated with the Pearson correlation (autoscaled variance/covariance matrix) to obtain the best visualization of the data in the PCA plot [26]. Instead, the PCA for the sensory descriptors was built on the cantered variance/covariance matrix (without variable scaling).

Additionally, one-way and two-way ANOVA (with interactions) on Time and different vinification (V1 and V2) were applied in order to study the variables most significant in terms of the factors. Tukey’s post-hoc tests for honestly significant difference (HSD) was also applied after the ANOVA modelling.

#### 4.5.2. Partial Least Square Regression (PLS)

Concerning the PLS models, the variables were centred and scaled for unit variance. Cross-validation with a Jack-knife approach (leave-one-out) [27] was used to validate the regression built on latent factors. For the selection of the optimal number of factors, a first PLS model was computed considering the number of components giving the minimum predicted residual error sum of squares statistics (PRESS). From this computation, it was further decided to recompute the model with the number of components showing the maximum Q^2^ cumulative index. This index, called cross-validation R^2^, was calculated using the formula (1):

(1) $$Q^{2}\mathrm{cum}\;\left(h\right)=1-\overset{h}{\underset{j=1}{\prod }}\frac{{\mathrm{PRESS}}_{\mathrm{kj}}}{{\mathrm{SSE}}_{k\left(j-1\right)}}$$

where h is the number of components used in the regression model, j is the observation and involves the PRESS statistics and Sum of Squares Errors (SSE). The maximization of Q^2^ index is equivalent to finding the most stable model [27]. The other two important indexes for understanding the quality of the model used were R^2^Y, R^2^X indexes, and VIPs. The R^2^Y index is the sum of the determination coefficients between the dependent variables and the h is first components measuring the explanatory power of the dependent variables in the model. The Variables Important in Projection (VIPs) is an index measuring the importance of the explanatory variables to build the t components (which are the principal component for the X matrix and Y matrix). The formula is (2):

(2) $${\mathrm{VIP}}_{\mathrm{hj}}=\sqrt{\frac{p}{\sum_{i=1}^{h}Rd\left(Y,t_{i}\right)}\cdot \overset{h}{\underset{i=1}{\sum }}w_{ij}^{2}Rd\left(Y,t_{i}\right)}$$

where *Rd(Y, t_i_)* is the redundancies which are defined as the mean of the squares of the correlation coefficients between variables and components. VIPs scores shown by the bar chart of the VIP (example at paragraph 2.3.1), where VIPs greater than 0.8 identify those variables that have the highest influence on the model. The last charts to consider for understanding the quality of the model are those of (i) residuals versus dependent variables (for the prediction and residuals), (ii) distance between the predicted and observed values and, (iii) bar charts of residuals. The maximum Q^2^ obtained was used for computing another PLS with the best quality for Q^2^ cumulated and R^2^Y and R^2^X [28].

#### 4.5.3. Principal Component Regression (PCR)

PCR was applied on each specific descriptor studied (as y-vector). Each PCR was computed arbitrarily with 15 components in order to define the best component number to be used in the definitive model. R^2^ parameter was also applied to decide how many components should be used in the model. R^2^ was calculated as (3):

(3) $$R^{2}=\frac{\sum_{i=1}^{n}w_{i}{\left({\hat{y}}_{i}-{\overset{ˉ}{y}}_{i}\right)}^{2}}{\sum_{i=1}^{n}w_{i}{\left(y_{i}-{\overset{ˉ}{y}}_{i}\right)}^{2}}\;\mathrm{with}\;\overset{ˉ}{y}=\frac{1}{W}\overset{n}{\underset{i=1}{\sum }}w_{i}y_{i}$$

where y is the observed value, $\hat{y}$ is the model predicted value, and $\overset{ˉ}{y}$ are the mean of observations. R^2^ is interpreted as the portion of the variability of the dependent y-variable explained by the model. The nearer R^2^ is to 1, the better is the model’s fitting of the data. It is important to note that this index depends on the number of variables in the model, for that reason another index (adjusted $\hat{R}$^2^) is widely used, which is a corrected R^2^, defined as (4):

(4) $${\hat{R}}^{2}=1-\left(1-R^{2}\right)\frac{W-1}{W-p-1}$$

where *W* is the number of all variables and *p* is the number of vectors. Another important value used for understanding the quality of the model is RMSE (root means square error) [26].

## Figures and Tables

**Table 1:** Quality index (Adj.-R^2^ and RMSE) of the PCR for olfactory descriptors on aroma compounds.

	Floral	Apple	Pear	Tropical Fruit	Dried Fruit	Fresh Vegetative	Spicy	Cleanness	Off-Odours
**Adj.-R^2^**	0.552	0.725	0.550	0.455	0.779	0.647	0.681	0.696	0.687
**RMSE**	0.339	0.312	0.509	0.523	0.260	0.403	0.214	0.134	0.171

**Table 2:** Quality index (Q^2^, R^2^Y, and R^2^X) for partial least square regression on visual and gustatory sensory descriptors.

Statistic	Comp.1	Comp.2	Comp.3	Comp.4	Comp.5	Comp.6
Q^2^ (cum)	0.241	0.239	0.252	0.332	0.347	0.347
R^2^Y (cum)	0.352	0.480	0.553	0.681	0.728	0.761
R^2^X (cum)	0.180	0.378	0.634	0.703	0.786	0.862

**Table 3:** Quality index (R^2^, standard deviation, RMSE) for PLS on visual and gustatory sensory descriptors.

	Yellow Colour	Sweetness	Sourness	Saltiness	Bitterness	Astringency	Warmness
R^2^	0.778	0.679	0.876	0.869	0.745	0.532	0.846
Std. deviation	0.395	0.267	0.215	0.263	0.339	0.269	0.399
RMSE	0.329	0.222	0.179	0.220	0.283	0.225	0.333

**Table 4:** List of descriptors and respective definitions used in the sensory analysis.

Descriptors	Definition
*Visual evaluation*	
Clarity	Absence of veiling and suspension in the wine
Colour tonality	Intensity of yellow colour
*Olfactory evaluation*	
Overall intensity	Total smell intensity perceived through the nose
Floral	Rose, elder aromas
Apple	Apple aroma
Pear	Pear aroma
Tropical fruit	Banana, pineapple, mango aromas
Dried fruit	Raisin, dried apricot, plum aromas
Fresh vegetative	Mint, sage aromas
Spicy	Liquorice, black pepper aromas
Cleanness	Absence of faults/taints odours
Off-odours	Presence of faults/taints odours
*Gustatory evaluation*	
Sweetness	Taste of sucrose
Sourness	Taste of tartaric acid solution
Saltiness	Taste of sodium chloride solution
Bitterness	Taste of caffeine solution
Astringency	Sensation related to drying in-mouth
Warmness	Sensation of alcohol (hot) in-mouth
Overall quality judgment	Objective assessment for the wine quality considering the sensory descriptors themselves

## Data Availability

Original data are available upon direct request to the corresponding author.

## Supplementary Materials

The following Supplementary materials are available online: Figure S1. Flowchart of the control (V1) and experimental (V2) winemaking procedures; Table S1. One-way ANOVA on the phenolic compounds for Time versus Retention Time (RT). Only the significant variables are shown according to Tukey HSD test (95% confidence). W: wine, 3, 6, 9, 12,18: months of storage in bottle. Different letters in the same column indicate significant differences evaluated by the post-hoc test; Table S2. One-way ANOVA on the non-volatile phenolic compounds for Wine versus Retention Time (RT). Only the significant variables are shown according to Tukey HSD test (95% confidence). V1: vinification without maceration, V2: vinification with maceration. Different letters in the same column indicate significant differences evaluated by the post-hoc test.; Table S3. Two-way ANOVA on the non-volatile phenolic compounds for Wine*Time versus Retention Time. Only the significant variables are shown according to Tukey HSD test (95% confidence). W: wine, 3, 6, 9, 12,18: months of storage in bottle, 1: wine without maceration, 2: wine with maceration. Different letters in the same column indicate significant differences evaluated by the post-hoc test; Table S4. One-way ANOVA on the volatile compounds for the Time. Only the significant variables are shown according to Tukey HSD test (95% confidence). W: wine, 3, 6, 9, 12,18: months of storage in bottle. Different letters in the same column indicate significant differences evaluated by the post-hoc test; Table S5. One-way ANOVA on the volatile compounds for Wine versus winemaking technique. Only the significant variables are shown according to Tukey HSD test (95% confidence). V1: vinification without maceration, V2: vinification with maceration. Different letters in the same column indicate significant differences evaluated by the post-hoc test; Table S6. Two-way ANOVA on the volatile compounds for Wine*Time versus RT. Only the significant variables are shown according to Tukey HSD test (95% confidence). W: wine, 3, 6, 9, 12,18: months of storage in bottle, 1: wine without maceration, 2: wine with maceration. Different letters in the same column indicate significant differences evaluated by the post-hoc test; Table S7. One-way ANOVA on the sensory descriptors for the Time. Only the significant variables are shown according to Tukey HSD test (95% confidence). W: wine, 3, 6, 9, 12,18: months of storage in bottle. Different letters in the same column indicate significant differences evaluated by the post-hoc test; Table S8. Two-way ANOVA on the sensory descriptors for the Wine*Time. Only the significant variables are shown according to Tukey HSD test (95% confidence). W: wine, 3, 6, 9, 12,18: months of storage in bottle, 1: wine without maceration, 2: wine with maceration. Different letters in the same column indicate significant differences evaluated by the post-hoc test. Only variables with significant differences are shown; Table S9. Tentative identification of volatile compounds with respective Retention Time (RT) and calculated Retention Index (RI) [13]; Figure S2. Standardized coefficients and standardized residuals/observations graphs of principal component regression for sensory descriptors. The coefficients are ordered by the *p*-value from the lowest to the highest and the rectangular shape indicate the level of significance (α = 0.001, 0.01 and 0.05). Aroma compounds labels are explicated in Table 2. A plot = standardized coefficients for Floral descriptor, B plot = standardized residuals for Floral descriptor, C plot = standardized coefficients for Apple descriptor, D plot = standardized residuals for Apple descriptor, E plot = standardized coefficients for Pear descriptor, F plot = standardized residuals for Pear descriptor, G plot = standardized coefficients for Tropical Fruit descriptor, H plot = standardized residuals for Tropical Fruit descriptor, I plot = standardized coefficients for Dried Fruit descriptor, L plot = standardized residuals for Dried Fruit descriptor, M plot = standardized coefficients for Fresh Veget. descriptor, N plot = standardized residuals for Fresh Veget. descriptor, O plot = standardized coefficients for Spicy descriptor, P plot = standardized residuals for Spicy descriptor, Q plot = standardized coefficients for Cleanness descriptor, R plot = standardized residuals for Cleanness descriptor, S plot = standardized coefficients for Off-odour descriptor, T plot = standardized residuals for Off-odour descriptor; Table S10. Tentative identification of non-volatile compounds for the HPLC analysis [13]; Figure S3. Standardized residuals graph of Partial Least Square regression (PLS) for the visual and gustatory descriptors. A: yellow color, B: sweetness, V = wine (V1 and V2).
