# Supplementary material for: Chemosensory Profile of South Tyrolean Pinot Blanc Wines: A Multivariate Regression Approach
Source: Molecules. 2021 Oct 15;26(20):6245. doi: 10.3390/molecules26206245 (PMC8538629; doi:10.3390/molecules26206245)
Supplement: Supplementary file 1 [file molecules-26-06245-s001.zip › molecules-1377609-supplementary.pdf]

Supplementary material

Chemosensory Profile of South Tyrolean Pinot Blanc Wines: A Multivariate Regression Approach

Simone Poggesi [1,2], Amanda Dupas de Matos [3,4], Edoardo Longo [1,2]*, Danila Chiotti [5], Ulrich Pedri [5], Daniela Eisenstecken [5], Peter Robatscher [5], Emanuele Boselli [1,2]

1 Faculty of Science and Technology, Free University of Bozen-Bolzano, Piazza Università 5, 39100 Bolzano, Italy; simone.poggesi@natec.unibz.it; emanuele.boselli@unibz.it; edoardo.longo@unibz.it
2 Oenolab, NOI Techpark, via Alessandro Volta 13, 39100 Bolzano BZ, Italy
3 Food Experience and Sensory Testing (Feast) Lab, Massey University, Palmerston North 4410, New Zealand; a.dupasdematos@massey.ac.nz
4 Riddet Institute, Massey University, Palmerston North 4410, New Zealand; a.dupasdematos@massey.ac.nz
5 Laimburg Research Centre, Laimburg 6, Pfatten (Vadena), 39040 Auer, Italy; danila.chiotti@laimburg.it; ulrich.pedri@laimburg.it; daniela.eisenstecken@laimburg.it; peter.robatscher@laimburg.it;
*Correspondence to: edoardo.longo@unibz.it; ORCID ID: 0000-0002-0594-6722

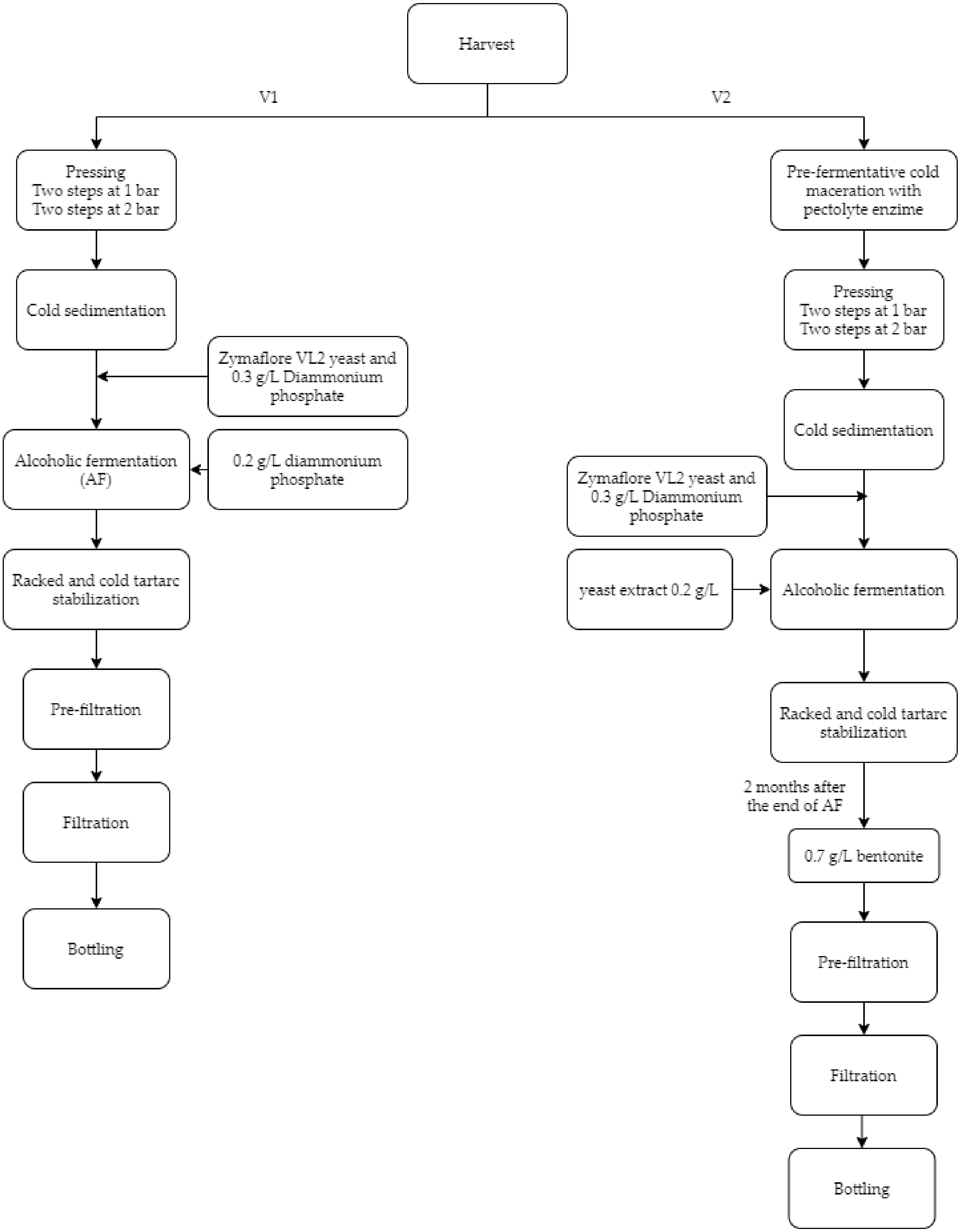

Figure S1. Flowchart of the control (V1) and experimental (V2) winemaking procedures.

Table S1. One-way ANOVA on the phenolic compounds for Time *versus* Retention Time (RT). Only the significant variables are shown according to Tukey HSD test (95% confidence). W: wine, 3, 6, 9, 12,18: months of storage in bottle. Different letters in the same column indicate significant differences evaluated by the *post-hoc* test.

**Time\RT**	**3.89**	**4.47**	**4.69**	**5.8**	**7.26**	**12.88**	**16.75**	**17.36**	**21.9**	**22.24**	**23.39**
W9	3288.1 a	20438.1 b	17628.8 b	1702.0 a	18341.7 ab	56919.5 ab	49699.3 a	61300.3 a	8896.0 b	25465.2 b	11188.3 a
W3	1800.0 a	30526.2 c	4206.0 a	5903.1 b	5955.2 a	64403.7 b	49376.3 a	42753.3 a	5247.5 a	16231.0 ab	22100.7 b
W6	8441.0 b	20197.9 b	34321.4 c	10087.0 c	19235.0 ab	47547.3 a	63696.2 b	40103.2 a	5228.2 a	17800.8 ab	23030.0 b
W18	506.0 a	18052.3 ab	18989.8 b	4599.8 b	36395.5 b	58064.5 ab	47620.3 a	59558.7 a	3763.3 a	7555.5 a	9914.2 a
W12	1232.5 a	12401.8 a	22580.2 b	6172.5 b	11905.0 ab	63430.8 b	50940.7 a	60612.7 a	2756.5 a	24259.7 b	11338.2 a
Pr > F(Model)	<0.0001	<0.0001	<0.0001	<0.0001	0.0	0.0	0.0	0.0	<0.0001	0.0	<0.0001

**Time\RT**	**24.07**	**24.75**	**26.17**	**26.63**	**27.24**	**36.35**	**41.74**	**45.22**	**47.49**	**48.46**	**50.22**	**51.81**
W9	22464.5 b	97200.2 ab	6191.3 abc	2169.7 b	16021.7 b	46069.0 b	21531.3 a	46888.3 ab	8035.3 a	6430.5 b	17323.5 a	7822.7 bc
W3	14291.7 ab	125611.3 b	9823.5 c	1760.4 ab	11376.3 ab	19658.0 a	25755.7 b	42937.3 a	13788.3 b	5988.8 b	17748.7 a	2581.3 a
W6	8977.3 a	92611.2 a	6427.3 bc	1774.5 ab	7772.8 a	20637.3 a	23537.3 ab	44769.8 ab	12114.3 b	6314.5 b	18630.8 a	4070.2 ab
W18	22056.2 b	99486.3 ab	2257.5 a	1099.3 a	11176.7 ab	26063.5 a	25006.3 b	52230.7 ab	5351.7 a	9414.8 c	88341.8 b	9627.3 c
W12	21887.3 b	101610.2 ab	3065.5 ab	1871.5 ab	12797.2 ab	15798.2 a	24992.0 b	59852.3 b	8249.5 a	2507.3 a	3992.2 a	7330.2 bc
Pr > F(Model)	0.0	0.0	<0.0001	0.0	0.0	<0.0001	0.0	0.0	<0.0001	<0.0001	0.0	0.0

Table S2. One-way ANOVA on the non-volatile phenolic compounds for Wine *versus* Retention Time (RT). Only the significant variables are shown according to Tukey HSD test (95% confidence). V1: vinification without maceration, V2: vinification with maceration. Different letters in the same column indicate significant differences evaluated by the *post-hoc* test.

**Wine\RT**	**6.37**	**6.54**	**7.26**	**7.72**	**10.5**	**12.44**	**12.88**	**20.3**	**23.39**
V1	13303.55791	103084.6667	25395.46667	11014.93333	34770.8	107792.2667	61681.13333	10977.6448	19075.13333
V2	21701.15975	63514.2	11337.46667	27229.73333	13467.13333	92370.06667	54465.2	14281.77206	11953.4
Pr > F(Model)	1.13974E-13	0.000488579	0.023403043	1.36515E-07	2.0135E-13	4.70443E-05	0.033348646	0.004016983	0.004127918

**Wine\RT**	**24.07**	**24.75**	**26.17**	**26.63**	**27.95**	**29.34**	**31.08**	**32.81**	**33.72**
V1	13809.06667	93030.4	4080.066667	2052.611729	250679.2	19873.46667	55279.26667	38959.8	48289.6
V2	22061.73333	113577.2667	7026	1417.552201	508562.1333	38061.4	97961.13333	76934.46667	120903.6667
Pr > F(Model)	0.000878166	0.003021913	0.018461931	0.002287998	2.44218E-18	5.69089E-16	1.40708E-15	8.07304E-08	1.75768E-11

**Wine\RT**	**36.88**	**39.95**	**45.22**	**49.63**	**50.22**	**51.81**	**52.65**	**53.04**
V1	15005.95698	2416.466667	41192.06667	13579.29003	11939.4	4461	3039.333333	767.4756234
V2	31684.43425	9363.2	57479.33333	135377.113	46475.4	8111.666667	7119.933333	5753.476344
Pr > F(Model)	8.89444E-11	1.28499E-12	1.79411E-06	7.0476E-21	0.027673707	0.002900883	0.005857537	1.35533E-08

Table S3. Two-way ANOVA on the non-volatile phenolic compounds for Wine*Time *versus* Retention Time. Only the significant variables are shown according to Tukey HSD test (95% confidence). W: wine, 3, 6, 9, 12,18: months of storage in bottle, 1: wine without maceration, 2: wine with maceration. Different letters in the same column indicate significant differences evaluated by the *post-hoc* test.

**Wine*Time\RT**	**3.89**	**4.47**	**4.69**	**5.8**	**6.37**	**6.54**	**7.26**
W3*2	1854.3 a	25385.7 c	4187.3 a	6686.1 bc	21089.4 bc	71198.7 abc	654.7 a
W6*2	9007.3 b	18448.5 abc	27217.5 c	11401.7 d	20337.0 b	40767.7 a	18524.3 a
W9*2	299.0 a	18552.1 abc	14288.7 ab	1662.0 a	23318.3 bc	57391.7 ab	18536.0 a
W18*2	513.0 a	17372.7 abc	14571.3 ab	5503.0 b	24024.2 c	71395.0 abc	14721.7 a
W12*2	1103.0 a	10212.7 a	19014.7 bc	6352.3 bc	19736.8 b	76818.0 bc	4250.7 a
W3*1	1745.7 a	35666.7 d	4224.7 a	5120.1 b	13825.1 a	113182.0 d	11255.7 a
W9*1	6277.2 b	22324.1 bc	20969.0 bc	1742.0 a	14079.3 a	160483.3 e	18147.3 a
W6*1	7874.7 b	21947.3 bc	41425.3 d	8772.3 cd	11876.3 a	69247.7 abc	19945.7 a
W12*1	1362.0 a	14591.0 ab	26145.7 c	5992.7 bc	13098.8 a	93899.7 cd	19559.3 a
W18*1	499.0 a	18732.0 abc	23408.3 bc	3696.7 ab	13638.2 a	78610.7 bcd	58069.3 b
Pr > F(Model)	<0.0001	<0.0001	<0.0001	<0.0001	<0.0001	<0.0001	0.0

**Wine*Time\RT**	**7.72**	**10.5**	**12.44**	**12.88**	**21.9**	**22.24**	**23.39**
W3*2	34653.3 d	6570.3 a	96755.7 ab	60633.7 ab	6105.9 ab	14567.7 ab	18002.0 cd
W6*2	17968.0 abc	11397.7 ab	91645.7 ab	44942.3 a	6095.9 ab	17585.7 ab	19655.0 d
W9*2	22606.0 bcd	14950.7 bc	94825.3 ab	54184.0 ab	9141.0 b	23061.7 ab	8004.0 ab
W18*2	31286.3 cd	20240.0 c	84670.0 a	54313.7 ab	5308.7 ab	6233.3 a	6726.7 a
W12*2	29635.0 cd	14177.0 bc	93953.7 ab	58252.3 ab	3195.7 a	22520.7 ab	7379.3 ab
W3*1	15636.3 ab	31562.0 d	114042.0 b	68173.7 b	4389.0 a	17894.3 ab	26199.3 e
W9*1	9258.0 ab	39460.0 e	107700.0 ab	59655.0 ab	8651.0 b	27868.7 b	14372.7 cd
W6*1	7767.0 a	35779.7 de	107695.3 ab	50152.3 ab	4360.5 a	18016.0 ab	26405.0 e
W12*1	9896.3 ab	31223.3 d	110007.7 ab	68609.3 b	2317.3 a	25998.7 ab	15297.0 cd
W18*1	12517.0 ab	35829.0 de	99516.3 ab	61815.3 ab	2218.0 a	8877.7 ab	13101.7 bc
Pr > F(Model)	<0.0001	<0.0001	0.0	0.0	<0.0001	0.0	<0.0001

**Wine*Time\RT**	**24.07**	**24.75**	**26.17**	**26.63**	**27.24**	**27.95**	**29.34**
W3*2	17380.7 cd	133964.7 c	12685.7 e	1488.6 abc	13062.0 ab	515144.7 b	41067.7 c
W6*2	11343.3 abc	108594.0 abc	8470.7 d	1495.5 abc	8015.7 a	511929.0 b	38261.3 bc

W9*2	28996.0 e	100966.7 abc	8234.0 cd	1699.7 abc	18929.7 b	508564.0 b	33589.3 b
W18*2	26188.7 e	112983.7 abc	2439.0 a	840.7 a	11302.7 ab	514768.3 b	39918.0 bc
W12*2	26400.0 e	111377.3 abc	3300.7 ab	1563.3 abc	13411.0 ab	492404.7 b	37470.7 bc
W3*1	11202.7 ab	117258.0 bc	6961.3 bcd	2032.3 abc	9690.7 ab	269719.0 a	20807.3 a
W9*1	15933.0 bcd	93433.7 ab	4148.7 ab	2639.7 c	13113.7 ab	238527.3 a	18067.7 a
W6*1	6611.3 a	76628.3 a	4384.0 abc	2053.5 abc	7530.0 a	259296.0 a	19252.3 a
W12*1	17374.7 bcd	91843.0 ab	2830.3 a	2179.7 bc	12183.3 ab	239239.7 a	18597.0 a
W18*1	17923.7 d	85989.0 ab	2076.0 a	1358.0 ab	11050.7 ab	246614.0 a	22643.0 a
Pr > F(Model)	<0.0001	0.0	<0.0001	0.0	0.0	<0.0001	<0.0001

**Wine*Time\RT**	**31.08**	**32.81**	**33.72**	**36.35**	**36.88**	**39.95**	**41.74**
W3*2	94549.7 b	109866.3 d	106488.3 bc	21442.0 ab	26144.0 bc	8321.7 b	26028.0 b
W6*2	102288.3 b	68278.3 bc	125859.3 c	23042.0 ab	35710.0 c	10855.3 b	24029.0 ab
W9*2	105071.0 b	65131.0 bc	124191.0 c	54874.0 d	31923.8 c	8746.0 b	22949.0 ab
W18*2	93399.0 b	71090.0 c	120366.0 c	31192.7 bc	30758.3 c	9234.7 b	25589.3 b
W12*2	94497.7 b	70306.7 c	127613.7 c	13115.0 a	33886.0 c	9658.3 b	25071.3 b
W3*1	50455.7 a	56996.0 b	38817.3 a	17874.0 a	12947.7 a	3536.0 a	25483.3 b
W9*1	59509.0 a	29470.7 a	48968.7 a	37264.0 c	14122.5 ab	1398.3 a	20113.7 a
W6*1	59733.7 a	32124.3 a	57590.0 ab	18232.7 a	15980.0 ab	1526.3 a	23045.7 ab
W12*1	53971.7 a	37479.7 a	61636.7 ab	18481.3 a	17364.7 ab	2893.7 a	24912.7 b
W18*1	52726.3 a	38728.3 a	34435.3 a	20934.3 ab	14615.0 ab	2728.0 a	24423.3 ab
Pr > F(Model)	<0.0001	<0.0001	<0.0001	<0.0001	<0.0001	<0.0001	0.0

**Wine*Time\RT**	**45.22**	**47.49**	**48.46**	**49.63**	**50.22**	**51.81**	**53.04**
W3*2	46516.7 bcd	14467.3 e	6363.0 b	144702.0 c	25679.7 e	2866.0 ab	5630.0 b
W6*2	53120.7 d	12661.3 de	6537.0 bc	142426.0 c	25806.7 e	4642.7 abc	7394.7 c
W9*2	54675.3 de	8856.0 bcd	6994.3 bc	134424.0 c	23604.0 de	11293.7 e	5624.4 b
W18*2	63374.3 ef	6439.7 ab	8986.0 cd	148942.6 c	155744.3 f	12262.3 e	8286.7 c
W12*2	69709.7 f	9711.7 bcd	2084.0 a	106391.0 b	1542.3 a	9493.7 de	1831.7 a
W3*1	39358.0 ab	13109.3 de	5614.7 b	20306.0 a	9817.7 bc	2296.7 a	242.7 a
W9*1	39101.3 ab	7214.7 abc	5866.7 b	11906.7 a	11043.0 c	4351.7 abc	876.6 a
W6*1	36419.0 a	11567.3 cde	6092.0 b	16124.7 a	11455.0 c	3497.7 ab	1060.1 a

W12*1	49995.0 cd	6787.3 ab	2930.7 a	5998.0 a	6442.0 b	5166.7 bc	239.3 a
W18*1	41087.0 abc	4263.7 a	9843.7 d	13561.1 a	20939.3 d	6992.3 cd	1418.7 a
Pr > F(Model)	<0.0001	<0.0001	<0.0001	<0.0001	<0.0001	<0.0001	<0.0001

Table S4. One-way ANOVA on the volatile compounds for the Time. Only the significant variables are shown according to Tukey HSD test (95% confidence). W: wine, 3, 6, 9, 12,18: months of storage in bottle. Different letters in the same column indicate significant differences evaluated by the *post-hoc* test.

TIME	ethyl butanoate	isoamyl alcohol	ethyl hexanoate	hexyl acetate	n-hexanol	acetic acid	ethyl nonanoate	isobutyl octanoate	2,3-butanediol (isomer 2)
W3	9629023.0 a	104570857.8 a	162258509.3 ab	6553991.2 b	2162873.0 a	2721137.0 a	6071838.5 ab	1359837.3 a	3172522.2 ab
W6	7176653.5 a	275041635.7 ab	222771644.8 b	6817206.2 b	4619098.8 ab	7112449.3 ab	4062815.6 a	1272712.4 a	2539418.0 a
W9	12830033.8 a	417002759.2 b	158783059.7 ab	4299457.8 ab	8070315.2 bc	4880609.3 a	5210349.2 a	1537794.9 ab	3030665.9 ab
W12	39258188.3 b	395073178.0 b	236195036.5 b	5522879.8 ab	8293809.5 c	12967871.0 b	8200115.3 ab	1946551.8 b	2835114.7 ab
W18	11482136.5 a	453909448.7 b	16648003.2 a	1679027.6 a	6874835.7 bc	1869598.9 a	14998974.5 b	1500399.7 ab	5026280.5 b
Pr > F(Model)	0.0	0.0	0.0	0.0	0.0	0.0	0.0	0.0	0.0

TIME	ethyl decanoate	diethyl succinate	n-decanol	citronellol	phenylethyl acetate	ethyl dodecanoate	isoamyl decanoate	butanedioic acid, ethyl isoamyl ester	ethyl tetradecanoate	octanoic acid
W3	514739828.8 ab	24056550.2 a	1378764.7 a	2292382.7 ab	9889275.0 bc	22796018.8 b	1593078.3 ab	2113721.0 ab	1844729.0 a	25287094.5 ab
W6	844466326.2 b	60505449.1 b	1605448.3 a	3129288.6 b	9127683.8 bc	26297094.7 b	1435522.3 ab	1407527.5 a	2954986.7 ab	28670641.3 ab
W9	386349003.5 ab	48594399.8 b	2144532.2 ab	1787446.4 ab	6052694.5 b	13033393.8 ab	1119831.9 a	1670046.3 ab	2281919.5 a	11640492.2 a
W12	672769804.3 b	83944718.0 c	3137022.2 b	3262661.7 b	10147861.7 c	25395182.3 b	1670410.2 ab	3227613.5 b	6470100.0 b	44674105.2 b
W18	1955693.5 a	85204167.7 c	1115411.0 a	671486.0 a	821468.7 a	1046472.7 a	1823650.5 b	2575638.0 ab	1252325.2 a	28471684.5 ab
Pr > F(Model)	0.0	<0.0001	0.0	0.0	<0.0001	0.0	0.0	0.0	0.0	0.0

Table S5. One-way ANOVA on the volatile compounds for Wine *versus* winemaking technique. Only the significant variables are shown according to Tukey HSD test (95% confidence). V1: vinification without maceration, V2: vinification with maceration. Different letters in the same column indicate significant differences evaluated by the *post-hoc* test.

WINE	isobutyl octanoate	2,3-butanediol (isomer 2)	octanoic acid
V1	1383650.4 a	3931887.9 b	37791269.7 b
V2	1663268.1 b	2709712.6 a	17706337.4 a
Pr > F(Model)	0.0	0.0	0.0

Table S6. Two-way ANOVA on the volatile compounds for Wine*Time *versus* RT. Only the significant variables are shown according to Tukey HSD test (95% confidence). W: wine, 3, 6, 9, 12,18: months of storage in bottle, 1: wine without maceration, 2: wine with maceration. Different letters in the same column indicate significant differences evaluated by the *post-hoc* test.

Wine*TIME	ethyl acetate	ethyl butanoate	isoamyl acetate	isoamyl alcohol	ethyl hexanoate	hexyl acetate
W6*1	153031895.3 e	11527325.3 ab	166152426.7 c	490121298.7 cd	374534741.0 f	10803738.3 d
W12*2	47255205.3 bc	28883200.0 ab	86189518.0 ab	481773448.0 cd	189224297.7 cd	4148764.7 abc
W12*1	57362912.3 bcd	49633176.7 b	84277660.0 ab	308372908.0 bc	283165775.3 e	6896995.0 bcd
W9*2	82743162.3 cd	17784800.7 ab	112955267.3 bc	533103264.0 d	191752996.7 cd	4833497.3 abc
W3*2	19850199.0 ab	11319695.5 ab	111687981.0 b	171224785.3 ab	213282255.7 d	7293433.0 cd
W18*1	89356227.3 d	10835088.7 ab	63984633.3 ab	485678554.7 cd	18936441.5 a	1685984.6 a
W18*2	64322600.0 cd	12129184.3 ab	52657089.3 a	422140342.7 cd	14359565.0 a	1672070.6 a
W3*1	8000205.3 a	7938350.4 ab	51828774.3 a	37916930.3 a	111234763.0 b	5814549.3 abc
W9*1	25580010.7 ab	7875267.0 ab	83502280.7 ab	300902254.3 bc	125813122.7 bc	3765418.3 abc
W6*2	7774152.0 a	2825981.7 a	38511891.0 a	59961972.7 a	71008548.7 ab	2830674.0 ab
Pr > F(Model)	<0.0001	0.0	<0.0001	<0.0001	<0.0001	<0.0001

Wine*TIME	n-hexanol	ethyl octanoate	acetic acid	isopentyl hexanoate	ethyl nonanoate	2,3-butanediol_1	isobutyl octanoate
W6*1	6276214.3 c	2601210139.3 b	12859486.0 de	3188272.0 c	1937586.3 ab	8781192.0 b	934589.3 a
W12*2	11154576.0 e	921327688.3 a	9574614.0 cd	1827692.9 ab	6322932.0 ab	4024661.7 a	2142457.7 c
W12*1	5433043.0 bc	534234478.5 a	16361128.0 e	2669352.3 bc	10077298.7 b	2290184.6 a	1750646.0 bc
W9*2	9781021.7 de	988151097.0 a	7526368.3 bc	1843773.5 ab	964719.7 a	3895508.7 a	1476147.6 abc
W3*2	3018469.0 ab	1045790439.0 a	2920123.0 ab	975214.7 a	7152031.2 ab	4081778.0 a	1478973.7 abc
W18*1	5651646.3 bc	574536756.0 a	1712576.2 a	1154698.0 a	23145237.7 c	2747458.3 a	1392873.3 abc
W18*2	8098025.0 cd	403834919.3 a	2026621.7 a	2064076.0 abc	6852711.3 ab	1981934.0 a	1607926.0 abc

W3*1	1307277.0 a	881745894.7 a	2522151.0 ab	1228653.0 a	4991645.9 ab	2537712.3 a	1240700.9 ab
W9*1	6359608.7 c	514423993.0 a	2234850.3 a	1141624.0 a	9455978.6 b	2901524.3 a	1599442.3 abc
W6*2	2961983.3 ab	464844756.0 a	1365412.7 a	889859.3 a	6188044.8 ab	2082089.3 a	1610835.4 abc
Pr > F(Model)	<0.0001	<0.0001	<0.0001	<0.0001	<0.0001	0.0	0.0

**Wine*TIME**	**n-octanol**	**2,3-butanediol_2**	**ethyl decanoate**	**diethyl succinate**	**n-decanol**	**citronellol**
W6*1	1732492.7 b	2552592.7 a	1389899738.7 f	61099394.7 bcde	2259411.7 ab	4602184.7 d
W12*2	1307119.7 ab	2559706.8 a	428472486.7 cd	93598986.7 e	3113406.0 b	3361627.0 cd
W12*1	831691.3 a	3110522.7 a	917067122.0 e	74290449.3 cde	3160638.3 b	3163696.3 bcd
W9*2	1430147.7 ab	2397446.3 a	547986787.7 d	58576764.7 bc	2084102.3 ab	2179687.3 abcd
W3*2	1304169.9 ab	3304293.8 a	453558567.0 cd	31504899.3 ab	2003707.7 ab	2148977.9 abcd
W18*1	1241543.4 ab	7291688.0 b	2279866.3 ab	92586411.0 de	1133988.2 a	776763.7 ab
W18*2	1100543.1 ab	2760873.0 a	1631520.7 a	77821924.3 cde	1096833.8 a	566208.3 a
W3*1	995915.0 ab	3040750.6 a	575921090.7 d	16608201.0 a	753821.7 a	2435787.5 abcd
W9*1	1132937.0 ab	3663885.5 a	224711219.3 abc	38612035.0 ab	2204962.0 ab	1395205.5 abc
W6*2	843902.3 a	2526243.3 a	299032913.7 bcd	59911503.6 bcd	951485.0 a	1656392.5 abc
Pr > F(Model)	0.0	<0.0001	<0.0001	<0.0001	0.0	0.0

**Wine*TIME**	**phenylethyl acetate**	**ethyl dodecanoate**	**isoamyl decanoate**	**butanedioic acid, ethyl isoamyl ester**	**phenylethyl alcohol**	**ethyl tetradecanoate**	**octanoic acid**
W6*1	12563510.0 e	39330586.7 c	1780219.9 a	1695571.7 a	27871428.7 ab	4793216.8 a	46765207.3 c
W12*2	7694521.7 bcd	22631257.3 abc	1831220.8 a	4742694.0 b	44293612.0 b	6038062.3 a	26807633.0 ab
W12*1	12601201.7 e	28159107.3 bc	1509599.7 a	1712533.0 a	30305257.7 ab	6902137.7 a	62540577.3 c
W9*2	6393279.7 bcd	15849198.0 ab	1092926.4 a	1757100.0 a	40406222.0 b	3096119.9 a	12484867.7 a
W3*2	9628360.3 cde	15412208.3 ab	1506264.3 a	2264945.5 a	16037198.3 a	2346290.7 a	26176018.7 ab
W18*1	864655.7 a	965967.3 a	2009717.7 a	2652980.3 ab	30454393.0 ab	1092043.9 a	44456276.7 bc
W18*2	778281.7 a	1126978.0 a	1637583.3 a	2498295.7 a	29723297.3 ab	1412606.6 a	12487092.3 a
W3*1	10150189.7 de	30179829.3 bc	1679892.3 a	1962496.5 a	30509252.8 ab	1343167.3 a	24398170.3 a
W9*1	5712109.4 bc	10217589.7 ab	1146737.3 a	1582992.7 a	27065153.7 ab	1467719.0 a	10796116.7 a
W6*2	5691857.7 b	13263602.7 ab	1090824.7 a	1119483.3 a	31243102.7 ab	1116756.7 a	10576075.3 a
Pr > F(Model)	<0.0001	<0.0001	0.0	0.0	0.0	0.0	<0.0001

Table S7. One-way ANOVA on the sensory descriptors for the Time. Only the significant variables are shown according to Tukey HSD test (95% confidence). W: wine, 3, 6, 9, 12,18: months of storage in bottle. Different letters in the same column indicate significant differences evaluated by the post-hoc test.

TIME	Clarity	Yellow color	Olfactory intensity	Floral	Apple	Pear	Tropical fruit
W3	8.9 b	4.3 b	6.4 b	4.2 a	4.4 a	4.1 a	3.7 a
W6	8.7 ab	4.6 b	6.2 b	4.9 a	4.7 ab	3.7 a	3.9 ab
W9	8.5 a	4.3 b	6.5 b	4.8 a	4.9 ab	4.3 ab	4.5 bc
W12	8.7 ab	3.5 a	4.4 a	4.8 a	5.3 bc	5.1 b	5.0 c
W18	8.4 a	3.2 a	4.2 a	4.2 a	5.7 c	4.0 a	3.3 a
Pr > F(Model)	0.0	<0.0001	<0.0001	0.0	<0.0001	0.0	<0.0001

TIME	Dried fruit	Spicy	Off-odor	Warmness	Sweetness	Sourness	Saltness	Bitterness	Overall judgement
W3	3.9 a	2.8 ab	1.6 ab	5.0 a	4.2 a	5.2 a	3.6 a	3.3 b	5.8 a
W6	4.3 ab	2.3 a	1.4 ab	5.3 ab	4.4 a	5.1 a	3.7 a	3.6 b	6.3 abc
W9	4.8 b	2.7 ab	1.4 ab	5.9 b	5.0 b	6.0 b	4.9 c	3.5 b	6.7 bc
W12	4.4 ab	2.2 a	1.2 a	5.6 ab	3.9 a	4.9 a	4.1 b	2.3 a	6.1 ab
W18	3.8 a	3.0 b	1.8 b	7.0 c	4.2 a	5.8 b	4.8 c	3.8 b	6.9 c
Pr > F(Model)	0.0	0.0	0.0	<0.0001	<0.0001	<0.0001	<0.0001	<0.0001	0.0

Table S8. Two-way ANOVA on the sensory descriptors for the Wine*Time. Only the significant variables are shown according to Tukey HSD test (95% confidence). W: wine, 3, 6, 9, 12,18: months of storage in bottle, 1: wine without maceration, 2: wine with maceration. Different letters in the same column indicate significant differences evaluated by the *post-hoc* test. Only variables with significant differences are shown.

Wine*TIME	Yellow color	Olfactory intensity	Floral	Apple	Pear	Tropical fruit
W9*1	4.8 d	6.4 b	5.3 b	4.8 abcd	4.2 a	4.9 bc
W9*2	3.8 abcd	6.7 b	4.3 ab	4.9 abcd	4.4 a	4.0 abc
W6*1	4.6 d	6.3 b	5.2 ab	4.8 abcd	3.9 a	4.0 abc
W18*1	2.9 a	4.3 a	4.1 a	5.9 d	4.4 a	3.3 a
W18*2	3.5 abc	4.2 a	4.3 ab	5.5 bcd	3.6 a	3.3 a
W12*1	3.2 ab	4.3 a	4.6 ab	5.6 cd	5.2 a	5.0 c
W3*1	4.5 cd	6.6 b	4.2 ab	4.5 ab	4.2 a	3.8 ab
W12*2	3.8 abcd	4.5 a	5.0 ab	5.0 abcd	5.0 a	5.0 c
W6*2	4.5 cd	6.0 b	4.7 ab	4.6 abc	3.5 a	3.7 a
W3*2	4.2 bcd	6.2 b	4.1 a	4.3 a	3.9 a	3.6 a
Pr > F(Model)	<0.0001	<0.0001	0.0	0.0	0.0	<0.0001

Wine*TIME	Spicy	Warmness	Sweetness	Sourness	Saltness	Bitterness	Overall judgement
W9*1	2.7 a	5.6 ab	5.0 b	6.0 c	4.9 de	3.5 bc	7.0 b
W9*2	2.8 a	6.2 bcd	5.0 b	6.0 bc	4.8 de	3.5 c	6.5 ab
W6*1	2.5 a	5.2 ab	4.3 ab	5.2 abc	3.6 ab	3.7 c	6.4 ab
W18*1	2.9 a	7.2 d	4.3 ab	5.8 abc	5.0 e	3.9 c	6.9 b
W18*2	3.1 a	6.9 cd	4.2 ab	5.9 abc	4.6 cde	3.8 c	6.9 b
W12*1	2.0 a	5.5 ab	4.0 a	4.8 a	4.3 bcd	2.5 ab	6.1 ab
W3*1	2.6 a	5.1 ab	4.1 a	5.3 abc	3.5 a	3.4 bc	6.0 ab
W12*2	2.4 a	5.8 abc	3.9 a	4.9 ab	4.0 abc	2.2 a	6.1 ab
W6*2	2.1 a	5.5 ab	4.6 ab	5.0 abc	3.7 ab	3.6 c	6.1 ab
W3*2	3.0 a	4.9 a	4.3 ab	5.1 abc	3.7 ab	3.3 bc	5.6 a
Pr > F(Model)	0.0	<0.0001	0.0	0.0	<0.0001	<0.0001	0.0

Table S9. Tentative identification of volatile compounds with respective Retention Time (RT) and calculated Retention Index (RI) [13].

Tentative compound identification	RT (min)	RI
ethyl acetate	5.2	795
ethyl butanoate	8.9	1033
isobutyl alcohol	11.0	1094
isoamyl acetate	11.9	1119
isoamyl alcohol	15.5	1207
ethyl hexanoate	16.6	1232
hexyl acetate	18.3	1271
n-hexanol	21.8	1352
ethyl octanoate	25.4	1436
acetic acid	26.0	1448
isopentyl hexanoate	26.4	1458
ethyl nonanoate	29.6	1535
2,3-butanediol	29.7	1573
isobutyl octanoate	30.3	1551
n-octanol	30.5	1556
ethyl decanoate	33.8	1638
isoamyl octanoate	34.5	1657
diethyl succinate	35.2	1674
ethyl 9-decenoate	35.8	1689
n-decanol	38.4	1759
citronellol	38.5	1762
phenylethyl acetate	40.4	1812
ethyl dodecanoate	41.4	1841
isoamyl decanoate	42.1	1860
ethyl isoamyl succinate	43.5	1898
phenylethyl alcohol	43.7	1905
ethyl tetradecanoate	48.2	2050
octanoic acid	48.4	2057

Figure S2. Standardized coefficients and standardized residuals/observations graphs of principal component regression for sensory descriptors. The coefficients are ordered by the *p*-value from the lowest to the highest and the rectangular shape indicate the level of significance (α = 0.001, 0.01 and 0.05). Aroma compounds labels are explicated in **Table 2**. A plot = standardized coefficients for Floral descriptor, B plot = standardized residuals for Floral descriptor, C plot = standardized coefficients for Apple descriptor, D plot = standardized residuals for Apple descriptor, E plot = standardized coefficients for Pear descriptor, F plot = standardized residuals for Pear descriptor, G plot = standardized coefficients for Tropical Fruit descriptor, H plot = standardized residuals for Tropical Fruit descriptor, I plot = standardized coefficients for Dried Fruit descriptor, L plot = standardized residuals for Dried Fruit descriptor, M plot = standardized coefficients for Fresh Veget. descriptor, N plot = standardized residuals for Fresh Veget. descriptor, O plot = standardized coefficients for Spicy descriptor, P plot = standardized residuals for Spicy descriptor, Q plot = standardized coefficients for Cleanness descriptor, R plot = standardized residuals for Cleanness descriptor, S plot = standardized coefficients for Off-odour descriptor,T plot = standardized residuals for Off-odour descriptor.

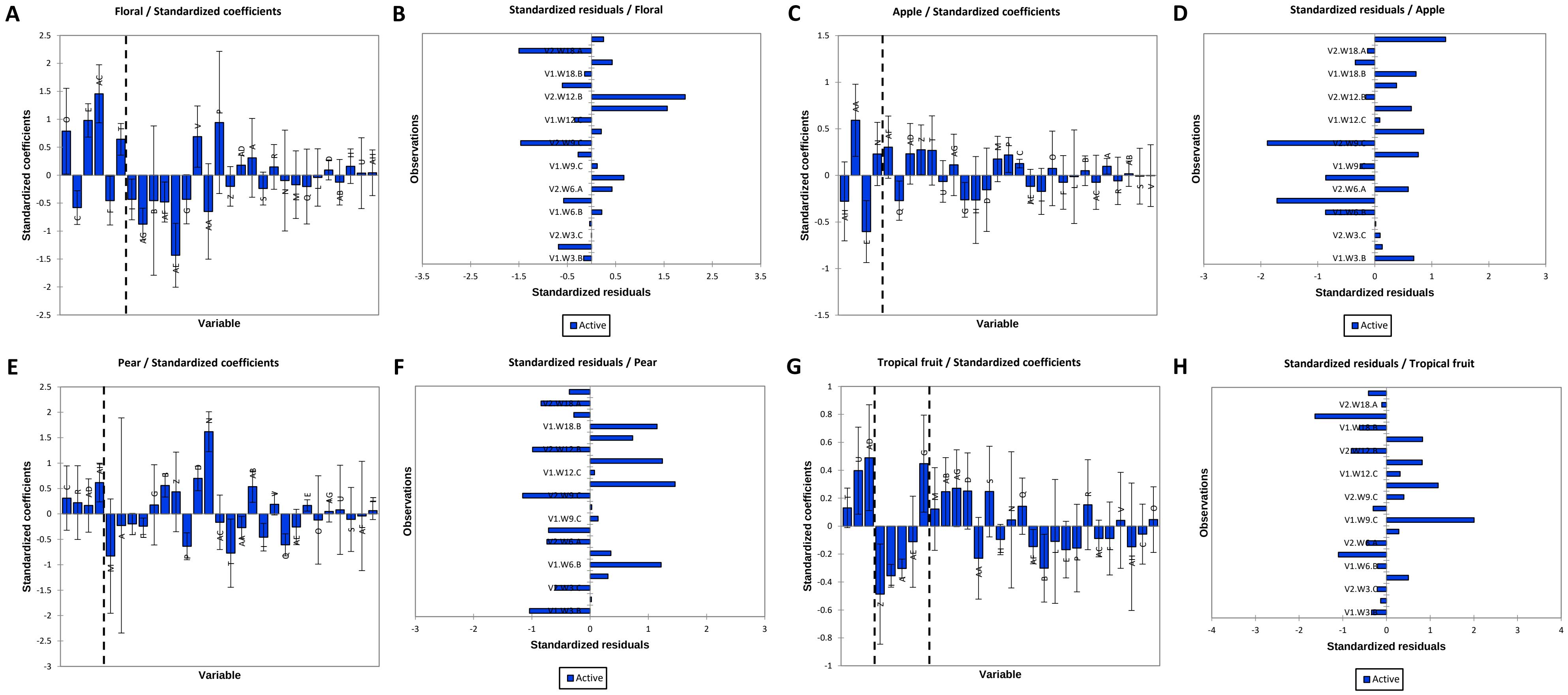

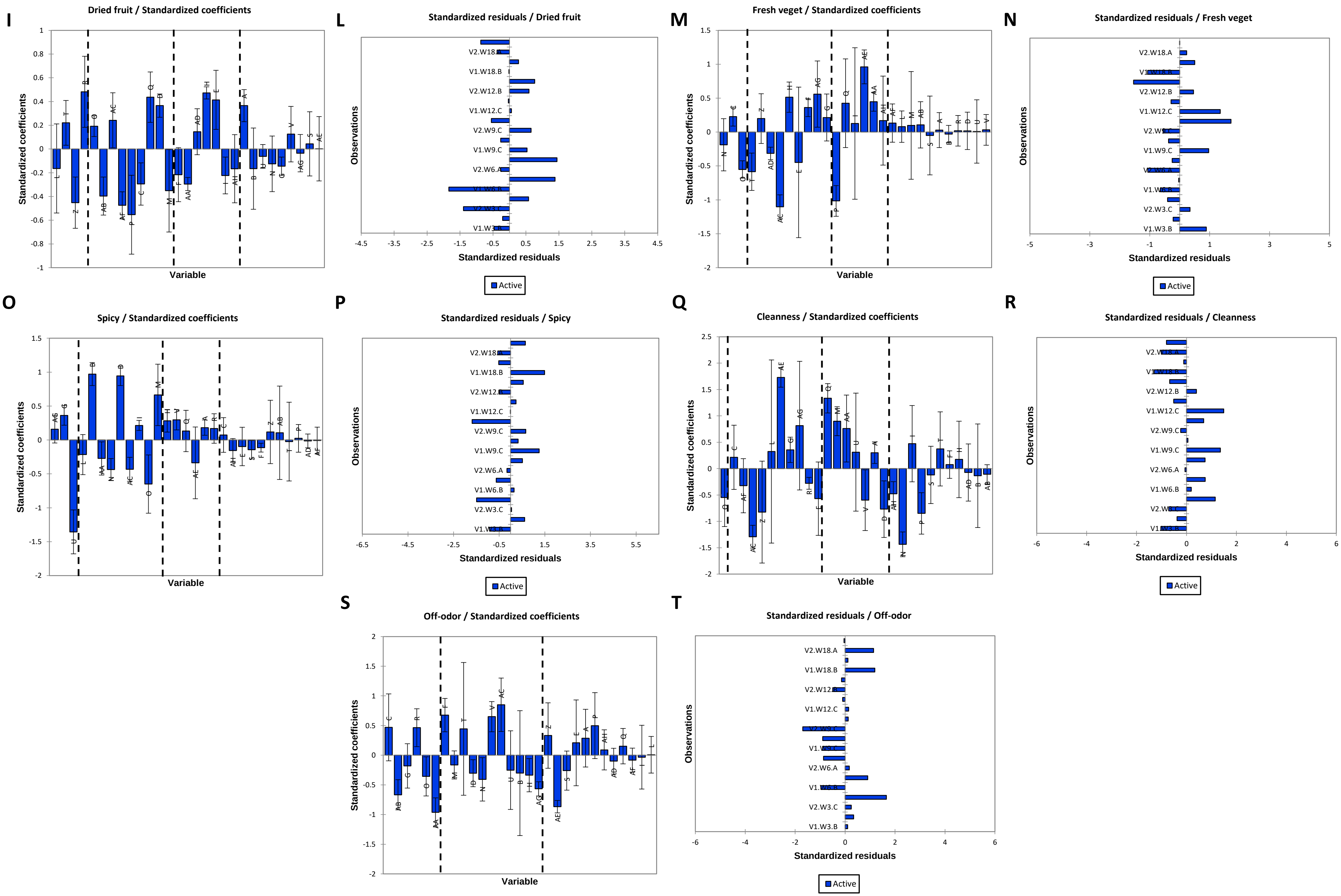
I
Dried fruit / Standardized coefficients
Standardized coefficients
Variable
L
Standardized residuals / Dried fruit
Observations
Standardized residuals
Active
M
Fresh veget / Standardized coefficients
Standardized coefficients
Variable
N
Standardized residuals / Fresh veget
Observations
Standardized residuals
Active
O
Spicy / Standardized coefficients
Standardized coefficients
Variable
P
Standardized residuals / Spicy
Observations
Standardized residuals
Active
Q
Cleanness / Standardized coefficients
Standardized coefficients
Variable
R
Standardized residuals / Cleanness
Observations
Standardized residuals
Active
S
Off-odor / Standardized coefficients
Standardized coefficients
Variable
T
Standardized residuals / Off-odor
Observations
Standardized residuals
Active

Table S10. Tentative identification of non-volatile compounds for the HPLC analysis [13].

Tentative compound identification	RT (min)	Wavelength (nm)	MS -	MS +
	3.9	~277	467.1	
	4.5	264		
	4.7	264	149.0	
	5.8	357		
	6.4	293		
	6.5	261		
	7.3	263		
	7.7	260	337.0	
	10.5	283	191.0	
	12.4	266	151.0	
	12.9	261	243.0	
Gallic acid	16.8	274	169.1	
	17.4	295		
	20.3	284		
	21.9	329	357	487
	22.2	328	143.1/161.1	
	23.4	265	382.1	
	24.1	278	315.1/378.1/477.2	
GRP	24.8	328	616.1	618.1
	26.2	320		
	26.6	310		
	27.2	275		
Caftaric acid	28.0	~296, 328	311.0/623.1	
	29.3	270	443.2/506.1	
Procyanidin dimer	31.1	275		579.1
Coutaric acid-cis	32.8	~290, 311	295.1, 163.1, 150.1	
Coutaric acid-trans	33.7	~290, 312	295.1, 163.1, 150.1	
Fertaric acid	36.4	329	325.1	
Caffeic acid	36.9	~290, 323	179.1	
	40.0	285		
Procyanidin dimer	41.7	278		579.1
Coumaric acid	45.2	~284, 308	163.1	
	47.5	~290	449.1	
	48.5	~290,317		487.1
Astilbin (isomer 1)	49.6	287	449.1/512.2(add.)	
Taxifolin	50.2	287	303.1	
	51.8	~295		
	52.7	~285		
Astilbin (isomer 2)	53.0	287	449.1/512.2(add.)	

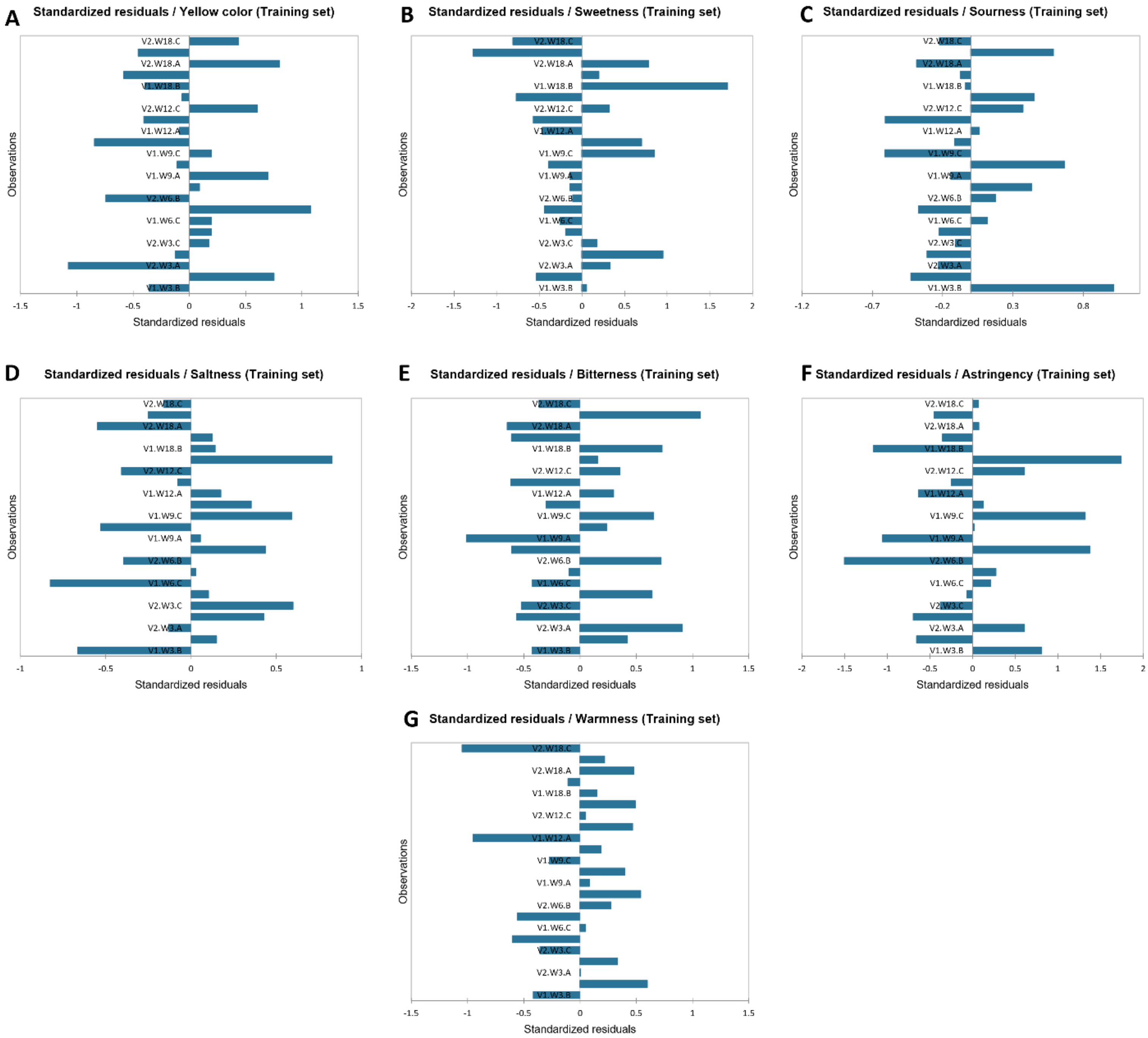

Figure S3. Standardized residuals graph of Partial Least Square regression (PLS) for the visual and gustatory descriptors. A: yellow color, B: sweetness, V = wine (V1 and V2),.
